# Conserved multi-tissue transcriptomic adaptations to exercise training in humans and mice

**DOI:** 10.1016/j.celrep.2023.112499

**Published:** 2023-05-12

**Authors:** Timothy M. Moore, Sindre Lee, Thomas Olsen, Marco Morselli, Alexander R. Strumwasser, Amanda J. Lin, Zhenqi Zhou, Aaron Abrishami, Steven M. Garcia, Jennifer Bribiesca, Kevin Cory, Kate Whitney, Theodore Ho, Timothy Ho, Joseph L. Lee, Daniel H. Rucker, Christina Q.A. Nguyen, Akshay T.S. Anand, Aidan Yackly, Lorna Q. Mendoza, Brayden K. Leyva, Claudia Aliman, Daniel J. Artiga, Yonghong Meng, Sarada Charugundla, Calvin Pan, Vida Jedian, Marcus M. Seldin, In Sook Ahn, Graciel Diamante, Montgomery Blencowe, Xia Yang, Etienne Mouisel, Matteo Pellegrini, Lorraine P. Turcotte, Kåre I. Birkeland, Frode Norheim, Christian A. Drevon, Aldons J. Lusis, Andrea L. Hevener

**Affiliations:** 1 Division of Cardiology, Diabetes, and Hypertension, Department of Medicine, David Geffen School of Medicine University of California, Los Angeles, Los Angeles, CA, USA; 2 Division of Endocrinology, Diabetes, and Hypertension, Department of Medicine, David Geffen School of Medicine University of California, Los Angeles, Los Angeles, CA, USA; 3 Department of Transplantation, Institute of Clinical Medicine, Faculty of Medicine, University of Oslo, Oslo, Norway; 4 Department of Nutrition, Institute of Basic Medical Sciences, Faculty of Medicine, University of Oslo, Oslo, Norway; 5 Department of Molecular, Cell and Developmental Biology, University of California Los Angeles, Los Angeles, CA, USA; 6 UCLA-DOE Institute for Genomics and Proteomics, University of California Los Angeles, Los Angeles, CA, USA; 7 Institute for Quantitative and Computational Biosciences – The Collaboratory, University of California, Los Angeles, Los Angeles, CA, USA; 8 Department of Chemical and Systems Biology, Stanford School of Medicine, Stanford, CA, USA; 9 Department of Biological Chemistry and Center for Epigenetics and Metabolism, University of California, Irvine, Irvine, CA, USA; 10 Molecular, Cellular, and Integrative Physiology Interdepartmental Program, University of California, Los Angeles, Los Angeles, CA, USA; 11 Department of Integrative Biology and Physiology, University of California, Los Angeles, Los Angeles, CA, USA; 12 Institute for Quantitative and Computational Biosciences, University of California, Los Angeles, Los Angeles, CA, USA; 13 Institute of Metabolic and Cardiovascular Diseases, UMR1297 Inserm, Paul Sabatier University, Toulouse, France; 14 Department of Biological Sciences, Dana & David Dornsife College of Letters, Arts, and Sciences, University of Southern California, Los Angeles, CA, USA; 15 Department of Human Genetics, University of California, Los Angeles, Los Angeles, CA, USA; 16 Department of Microbiology, Immunology and Molecular Genetics, University of California, Los Angeles, Los Angeles, CA, USA; 17 Iris Cantor-UCLA Women’s Health Research Center, Los Angeles, CA, USA; 18 Veterans Administration Greater Los Angeles Healthcare System, Geriatric Research Education and Clinical Center (GRECC), Los Angeles, CA, USA; 19 These authors contributed equally; 20 Lead contact

## Abstract

Physical activity is associated with beneficial adaptations in human and rodent metabolism. We studied over 50 complex traits before and after exercise intervention in middle-aged men and a panel of 100 diverse strains of female mice. Candidate gene analyses in three brain regions, muscle, liver, heart, and adipose tissue of mice indicate genetic drivers of clinically relevant traits, including volitional exercise volume, muscle metabolism, adiposity, and hepatic lipids. Although ~33% of genes differentially expressed in skeletal muscle following the exercise intervention are similar in mice and humans independent of BMI, responsiveness of adipose tissue to exercise-stimulated weight loss appears controlled by species and underlying genotype. We leveraged genetic diversity to generate prediction models of metabolic trait responsiveness to volitional activity offering a framework for advancing personalized exercise prescription. The human and mouse data are publicly available via a user-friendly Web-based application to enhance data mining and hypothesis development.

## In brief

Moore et al. studied over 50 traits before and after exercise intervention in middle-aged men and 100 diverse strains of female mice. Genetic diversity of the mouse panel was leveraged to generate a publicly available data repository that can be mined via a user-friendly Web-based application to enhance hypothesis development.

## Graphical Abstract

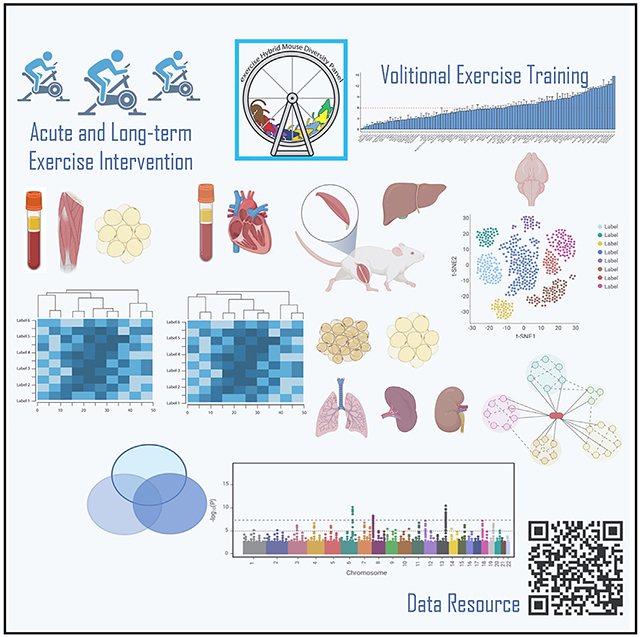

## INTRODUCTION

Physical activity is routinely prescribed by physicians to reverse or prevent complications associated with cardiometabolic disorders.^1,2^ Physical activity is one of the few clinical interventions effective at improving human health regardless of age,^3^ sex,^4^ ethnicity,^5^ or cardiometabolic status.^6^ In addition to cardiometabolic health, daily physical activity also reduces cancer incidence and recurrence,^7^ improves cognitive function and mental health, and protects against neurological disorders.^8^ Cardiorespiratory fitness, as assessed by maximum oxygen uptake (VO_2_ max), is reproducibly improved in mouse and human in response to increasing physical activity, and is one of the strongest predictors of all-cause mortality.^9-12^ Considering the rise in metabolic-related disease and overwhelming evidence supporting the health benefits of physical activity for mitigating chronic disease burden, it is concerning that long-term adherence to exercise prescription by the general population remains relatively poor.^13^ Thus, there is an urgent need to understand the molecular mechanisms improving metabolism as well as best practices for exercise prescription leading to greater adherence and health outcomes.

Because previous studies interrogating the benefits of exercise have been performed predominantly on a limited number of tissues and pathways, typically skeletal muscle and the cardiorespiratory system, we employed an unbiased assessment of whole animal trait and genome-wide responses to exercise training. We performed a large-scale project where mice from the Hybrid Mouse Diversity Panel (HMDP) performed voluntary wheel running for 30 days. The HMDP is a powerful and unique genetic resource including 100 diverse inbred strains of mice. We have previously used the HMDP to perform molecular dissection of complex cardiometabolic-related traits.^14-17^ The HMDP enables high-resolution genome-wide association studies (GWASs) and assessment of gene-by-environment interactions (e.g., physical activity). The HMDP has been particularly powerful when integrated with multi-omics analyses. In the current study, 12 tissues were harvested from each animal of the exercise (Exc) HMDP providing quantification of 50 distinct whole-body and tissue-specific physiological traits. Furthermore, we integrated our ExcHMDP data with the human Skeletal Muscle, Myokines and Glucose Metabolism (MyoGlu) study, including longitudinal collection of clinical traits and multiple biopsies of tissues following acute (one session of exercise training) and long-term (12 weeks) exercise intervention.^18,19^ MyoGlu and ExcHMDP are complementary datasets where both human and mouse tissue samples were subjected to multi-omics analyses, and these data were integrated with a variety of phenotypic traits.

Relationships between exercise workload and several cardiometabolic traits, including but not limited to tissue weights, plasma lipid levels, and insulin sensitivity, were examined. We performed global transcriptomic analyses of key metabolic tissues including skeletal muscle, white adipose, brown adipose, brain, liver, and heart, and integrated these data with phenotypic trait outcomes following exercise intervention. We identified mouse genetic factors underlying voluntary exercise behavior and the molecular and physiologic responses to exercise training. These findings have been made publicly available in a user-friendly and simple Web-based application designed to allow researchers the opportunity to visualize, compare, and interrogate data between mouse and human. Our study highlights the value of cross-species, multi-tissue genetic analyses and underscores the need for personalized prediction modeling to improve individual exercise prescription and adherence outcomes to mitigate disease risk.

## RESULTS

### Study design

To understand the role of genetics in exercise adaptation, we used a 100-strain panel of mice, the HMDP (Table S1A).^20^ HMDP strains are inbred, allowing for reliable comparisons between different HMDP studies. Thus, the HMDP is an expandable data resource. In our study, termed the exercise (Exc) HMDP, mice from each strain were randomly divided into two groups: exercise trained (TRN) or sedentary (SED) (Table S1A). Mice were allowed to exercise using an in-cage running wheel for 30 days, a sufficient time to induce exercise training adaptations.^21-23^ Following 30 d of exercise intervention, wheels were locked and mice were euthanized 30 h later to avoid the confounding effects of the last exercise bout.^24^ Mice were fasted during the final 6 h of the 30-h recovery period from the last exercise bout, to ensure a post-absorptive state. We examined the translational relevance of our studies in mice by integrating our findings with a longitudinal exercise intervention study including aerobic exercise as well as strength training, clinical parameters, and molecular measurements in human subjects. The MyoGlu study included 26 previously sedentary Norwegian men.^18,19^ The biopsy schedule allowed for the examination of tissue transcript response to acute (45 min of cycling, 70% VO_2_ max) and long-term exercise intervention (12 weeks, 4 × 60-min weekly sessions including strength and endurance training). An overview of the study design is provided in Figure S1A.

### Genetic regulation of voluntary wheel running

**Figure 1.**
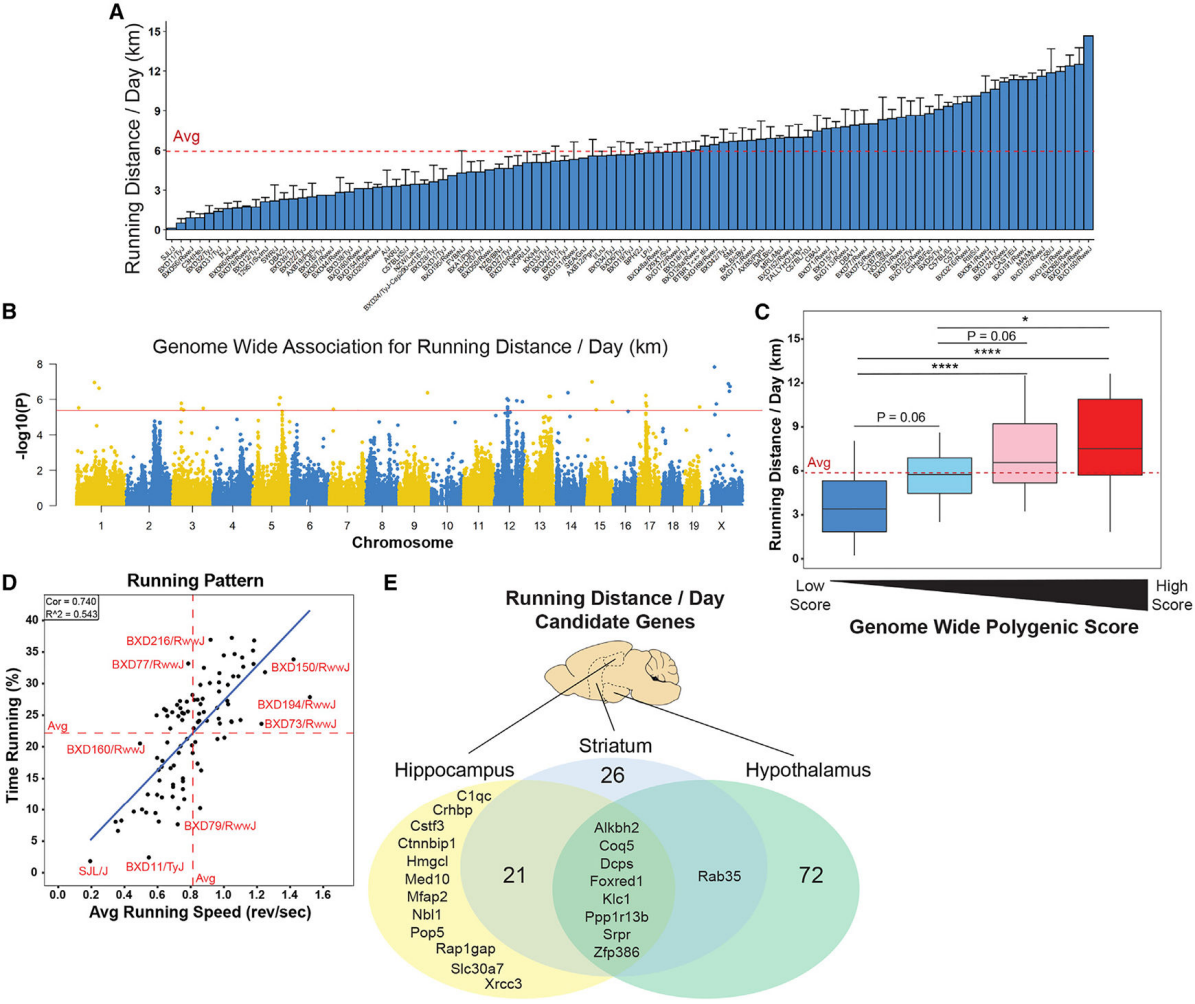
The impact of genetic variation and central regulators of voluntary exercise Female mice from the Hybrid Mouse Diversity Panel (HMDP) were trained (TRN) using in-cage running wheels or remained sedentary (SED) for 30 days. Transcriptomics data from the MyoGlu study of normal-weight and overweight individuals subjected to both acute and long-term endurance exercise were integrated for subsequent comparative analyses and made publicly available in a Shiny Web application. (A) Average daily running distance (km), dashed line indicates average. (B) GWAS for average daily running distance, solid line indicates significance threshold. (C) Genome-wide polygenic score for running distance per day (km) of strains stratified by quartile; *p < 0.05, ***p < 0.001, ****p < 0.0001. Only using SNPs with between *r*^2^ < 0.6 and GWAS p < 0.01. Dashed line indicates overall average. (D) Correlation of average running speed (revolutions/s) with percentage time running (% of 24-h period); solid blue line indicates least-squares regression line. Dashed lines indicate axis strain average. (E) Venn diagram showing overlap of candidate gene analysis for average daily running vs. previously published HMDP of RNA sequencing in hippocampus, hypothalamus, and striatum. Correlations were considered significant at FDR < 0.05.

We observed substantial strain dependent variation in the mean volitional daily running distance in TRN ExcHMDP mice (5.94 ± 3.43 km; Figure 1A), consistent with rodent exercise volume observed in other training studies.^25,26^ In addition to comparisons of running distance, running pattern could also affect the adaptation to daily activity. Thus, we determined the average running speed (revolutions/s) and percentage time running over each 24-h period. Running speed and time were strongly correlated variables (*R*^2^ = 0.54). The heritability of running distance, corresponding to the fraction of variance explained by genetics, was 0.68 ± 0.05 for the ExcHMDP.^27,28^

We performed a GWAS that revealed several significant loci (Figures 1B and 1C; Table S1B and S1C). As would be predicted, these data suggest that running distance is a polygenic trait. Polygenic traits can be assessed by examining the cumulative effect of separate single-nucleotide polymorphisms (SNPs).^29^ A genome-wide polygenic score was computed by summing the effect of each SNP from the GWAS of running distance (Figures 1B-1D and S2). Regardless of the computational method, as few as 100 SNPs were significantly associated with running distance and genome-wide polygenic score.

Although it is well documented that VO_2_ max, an index of exercise capacity,^30^ is largely regulated by the cardiorespiratory system,^31-34^ and volitional activity is arguably controlled by the CNS,^35,36^ we questioned whether prolonged voluntary wheel running could also be regulated by exercise capacity of the periphery. Mean running distance per day from the ExcHMDP was correlated with heart phenotypes from a prior sex- and age-matched HMDP.^37^ Of the nearly 30 heart phenotypes, none significantly correlated with running distance (p > 0.05; Figure S3A). We also conducted candidate gene identification analysis and found no candidate genes in cardiac tissue central in the regulation of daily running distance (false discovery rate [FDR] >0.05). These data suggest that voluntary wheel running distance is predominantly regulated by cardiac-independent factors in *Mus musculus*.

Because published findings suggest that ambulatory movement is driven by the CNS,^38^ we performed candidate gene identification analysis on three brain regions from prior age-matched mouse panels.^39,40^ Several potential candidate genes were identified in the three brain regions (FDR < 0.05; Figure 1E; Table S1B). The hypothalamus possessed the highest number of candidate genes (n = 81), followed by striatum (n =56) and hippocampus (n = 41). Several candidate genes were shared between all three brain regions (n = 8; Figures 1E and 1F). Thus, our findings suggest that voluntary wheel running is significantly controlled by all three brain regions studied: hypothalamus, striatum, and hippocampus.

Because the hypothalamus possessed the highest number of candidate genes regulating voluntary wheel running, hypothalamic single-cell RNA sequencing was performed on a separate cohort of sedentary and trained C57BL/6J mice that engaged in the same exercise protocol as the ExcHMDP animals. Seventeen distinct cell populations were identified in the hypothalamus (Figures S4A and S4C). Differential gene expression analysis within each cell population revealed significant differences in transcript abundance between the two groups, TRN vs. SED (FDR < 0.05; Figure S4B). The gene *mt-Rnr2,* encoding mitochondrial 16S rRNA, *Humanin,* was significantly increased in nearly all cell types. Recent research has linked *Humanin* expression to exercise intensity and aging-related diseases, including Alzheimer’s.^41-43^

### Leveraging genetic variation to predict physiological traits outcomes in response to exercise

**Figure 2.**
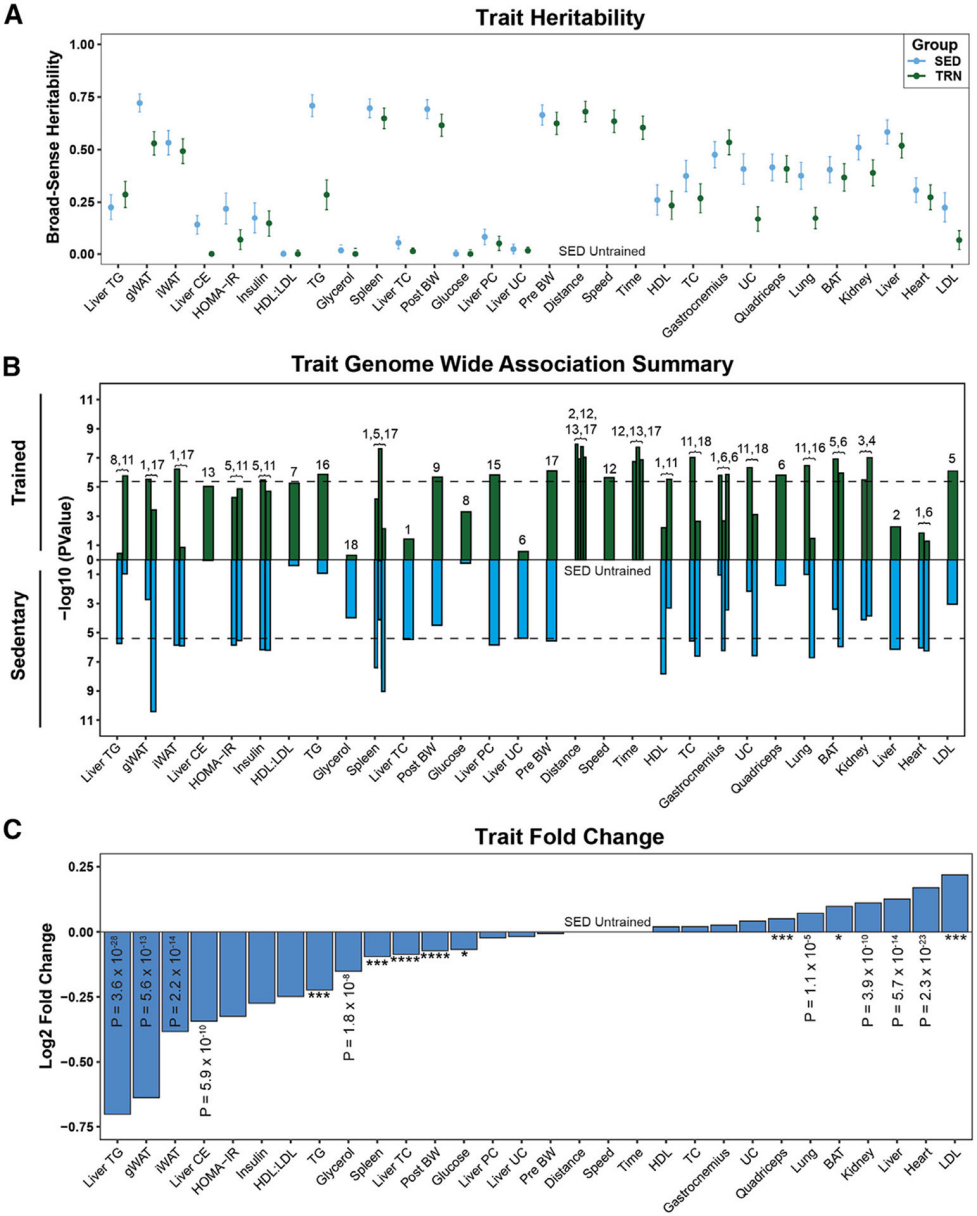
Trait heritability and the effect of genetic architecture on exercise adaptations (A) Heritability estimates of each trait for sedentary (blue) and exercise trained (green). (B) Top QTLs from GWAS of each trait in sedentary (blue) and exercise-trained (green) animals. Chromosome location of QTL indicated above bar. Dashed lines indicate significance threshold (−log_10_(p value)). (C) The log 2-fold change of traits across all strains relative to the sedentary group. *p < 0.05, ***p < 0.001, ****p < 0.0001.

**Figure 3.**
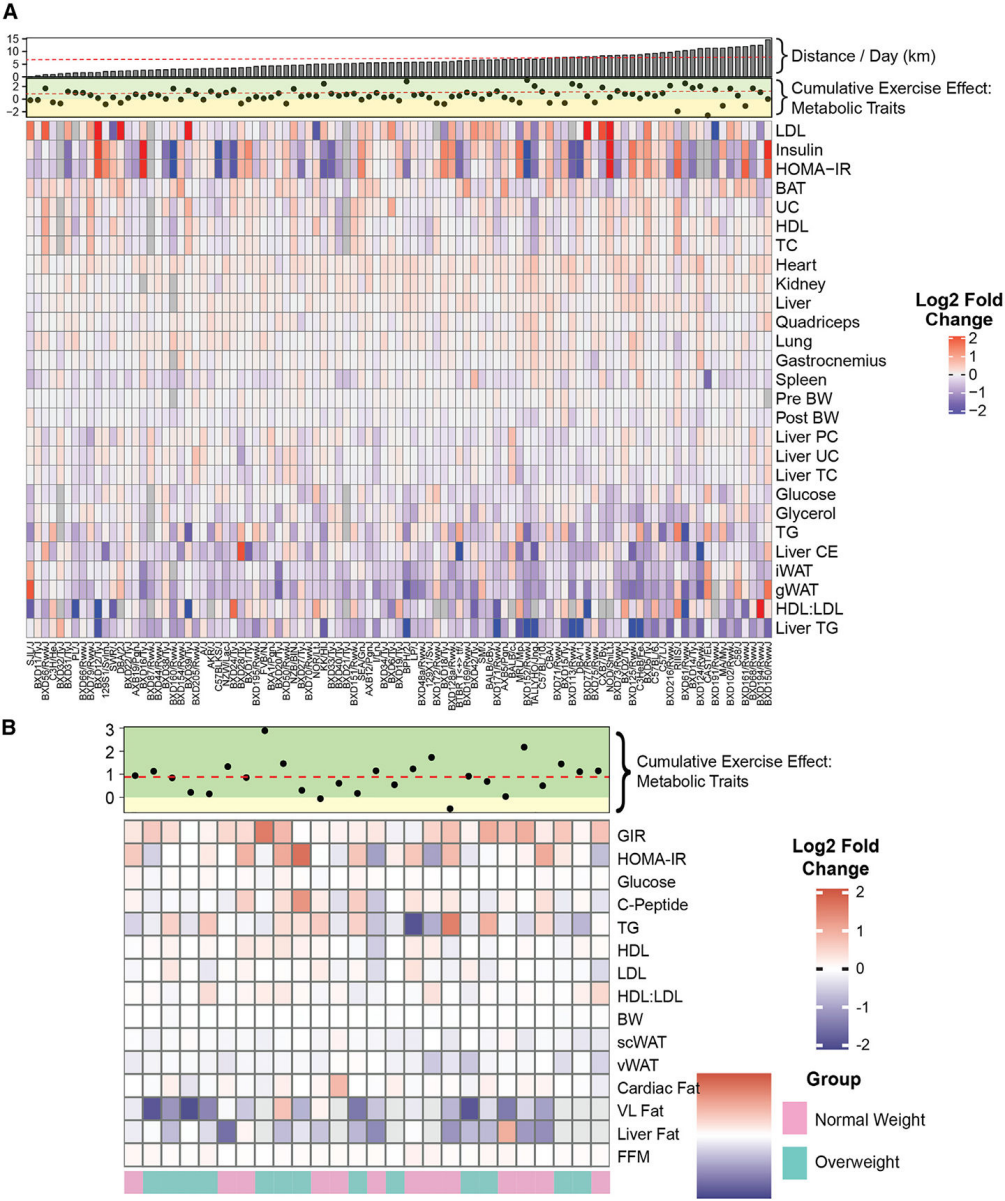
The effects of exercise on physiological trait outcomes (A and B) Fold change heatmap of each trait by strain (A, ExcHMDP) or group (B, MyoGlu). Positive or negative values indicate increase or decrease in exercise-trained mice or after exercise intervention in humans. Cumulative effect of exercise on metabolic traits with corresponding improved (green) or diminished (yellow) regions and distance run by mice per day plotted along the top. Dashed lines indicate average of variable.

A primary goal of the ExcHMDP was to examine the role of genetics in controlling physiological responses to exercise intervention. We calculated SNP heritability for each group (SED vs. TRN) for 50 physiological traits. SNP heritability was similar for most, but not all, traits between groups. Thus, group-specific GWAS was performed (Figure 2A; Table S1C), revealing unique as well as conserved loci for each trait in SED vs. TRN (p < 4.1 × 10^−6^; Figure 3B; Table S1D). The majority of traits showed group-specific quantitative trait loci (QTLs), indicating the presence of a QTL in one group but not the other. Of note, a specific 3-Mb region on Chr17 had a QTL for seven traits among both groups. These examples highlight a distinct physiology that is genetically regulated independent of exercise training status.

In response to exercise, the majority of traits were significantly different between TRN vs. SED (p < 0.05; Figure 2C). Exercise reduced liver and plasma lipids, adipose tissue mass, and blood glucose concentration, but increased heart, liver, and kidney mass. Because of the significant inter-strain variation in running distance, we determined whether running distance affected specific trait outcomes. If the correlation between SED and TRN for a trait is unchanged when distance is added as a covariate, then the trait response is largely independent of running distance. All correlations remained significant when running distance was added as a covariate (Table S1E). We then determined the mouse strain by group interaction. We observed that 85% of physiological traits have a significant interaction between mouse strain and group (SED vs. TRN) indicating a gene × exercise effect after adjustment for multiple comparisons (p < 0.05; Tables S1E and S1F).

Considering the differences in heritability estimates and phenotypic trait outcomes between the two groups, an unbiased principal-component analysis (PCA) was performed, revealing a significant difference between the groups, SED vs. TRN (p < 0.001; Figure S5A). Strains displayed a variable response to exercise independent of running distance. Similar to the ExcHMDP, MyoGlu subjects also displayed a variable response to exercise, although, in general, cumulative group differences (post exercise vs. pre-exercise) from the PCA analyses were statistically significant (p < 0.01; Figure S5B; Tables S1G-S1J). These findings highlight the importance of genetic architecture underlying exercise-induced adaptation.

Next, we determined the correlation structure among traits within a group (SED vs. TRN) from the ExcHMDP to identify salient trait-trait relationships. As expected, related traits showed strong within-group correlations (p < 0.01; Figure S5C). Daily running distance displayed significant correlations with traits that were also the most significantly different between groups (p < 0.01). Traits displaying the largest change in correlation structure were the most significantly different between the two groups, SED vs. TRN.

All assessed traits for ExcHMDP and MyoGlu studies are presented as a heatmap (Figures 3A and 3B). Because metabolic health is typically evaluated using multiple clinical parameters, we generated an index to reflect the cumulative metabolic effect of exercise in mice and humans. This metabolic index increased in 81% of strains following training (Figure 3A) but did not correlate with daily running distance (p > 0.05, *R*^2^ = 0.01), substantiating the notion that genetic architecture, in large part, drives physiological adaptation to exercise. Similar to rodents, the metabolic index was elevated in 90% of human subjects following exercise training intervention (Figure 3B). Leveraging genetic diversity by studying a 100-strain mouse panel as well as human subjects discordant for metabolic health and fitness allowed us to determine the importance of genetics vs. training volume in driving physiological responsiveness to daily activity.^44^

### Molecular responses to exercise in skeletal muscle

**Figure 4.**
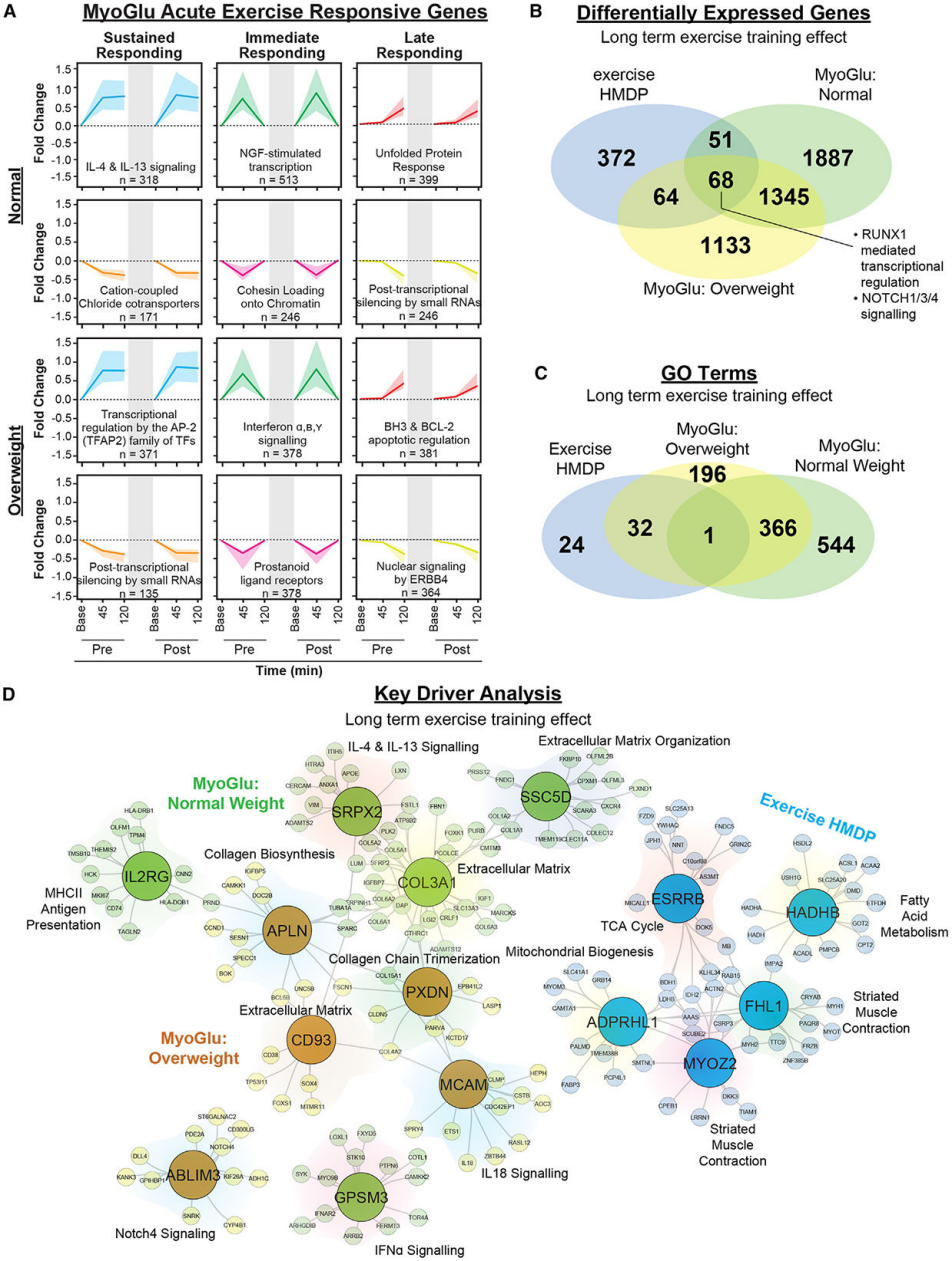
The impact of short-term and long-term exercise on transcript response and key drivers of physiological adaptation (A) Gene pattern identification from skeletal muscle of MyoGlu. Top two rows are normal weight and bottom two rows reflect data from overweight subjects. Left column indicates transcripts with sustained increase or decrease. Middle column indicates transcripts increasing or decreasing immediately after acute exercise only. Right column indicates transcripts increasing or decreasing 2 h after acute exercise cessation. Top GO term and gene set size indicated within. Dark colored line indicates fold change median for gene set. Shaded region around line indicates interquartile range of fold changes for gene set. Gray shaded region within each box indicates 12-week exercise intervention. (B and C) Venn diagram showing the overlap among groups for (B) DEGs or (C) Gene Ontology (GO) terms. MyoGlu are post baseline vs. pre-baseline for each group. (D) KDA of DEG colored by group. Key driver genes are enlarged within circles with the corresponding regulated genes within surrounding shaded region. Top GO term for each shaded region are indicated.

We explored whether exercise-responsive gene signatures in skeletal muscle were influenced by BMI for both acute and longer-term exercise intervention in previously sedentary men. In both normal-weight and overweight men of the MyoGlu trial, we identified sets of genes adapting in coordinated fashion following acute and chronic exercise (FDR < 0.05; Figure 4A; Table S1F). Acute exercise-responsive genes showed at least one of the following patterns: (1) sustained change following exercise that persisted 2 h post, (2) change immediately following exercise and returning to baseline 2 h post, or (3) change after the 2-h exercise recovery period only. Early-response genes reflected inflammatory and immune processes (e.g., *IL-4* and *IL-13*), whereas late-response genes were enriched for transcripts associated with apoptosis and the unfolded protein response. Transcripts decreasing in expression in response to acute exercise reflected gene silencing, chromatin folding, and transcriptional or translational regulation. We observed substantial overlap between normal-weight and overweight men within all acute exercise-responsive gene sets identified (average overlap of differentially expressed genes [DEGs] between groups = 46%; Figures S6A-S6F).

Moreover, we studied the long-term effects of exercise intervention on transcript expression in skeletal muscle of men in the MyoGlu trial. We identified transcripts changing after long-term exercise that were not significantly altered by acute exercise (described above) for both normal-weight and overweight men (FDR < 0.05; Figures S7A and S7D). Long-term exercise-responsive transcript signatures displayed less overlap between groups (normal weight vs. overweight) compared with acute exercise-responding transcript sets (average overlap of DEGs between groups = 16%; Figures S6G and S6H; Tables S1F-S1G). In normal-weight men, exercise increased transcripts associated with processes related to the immune system and inflammation (FDR < 0.05, Figure 6B). Transcripts with decreased expression in response to exercise intervention for normal-weight men were enriched for mRNA regulation, protein assembly, and post-translational modification (FDR < 0.05; Figure S7C). In overweight men, the response was markedly different: transcripts increased in expression were enriched for mitochondrial phenotypes and fatty acid metabolism (FDR < 0.05; Figure S7E). Transcripts related to glycolysis and gluconeogenesis were reduced in muscle expression among overweight men (Figure S7F). Increases in transcripts enriched for nervous system development, extracellular matrix, and angiogenesis were observed in both groups, whereas transcripts associated with DNA repair, organelle protein transport, transcriptional processes, and macroautophagy were reduced in both groups following exercise intervention.

Similar to overweight men, mice from the ExcHMDP showed an enrichment for metabolic processes including fatty acid β-oxidation, pyruvate metabolism, mitochondrial membrane transport, and electron transport (FDR < 0.05; Figure S8). Approximately 33% of transcripts significantly changed in mice were also changed in humans following long-term exercise training (FDR < 0.05; Figure 4B). These DEGs transcending species and biological sex were significantly enriched for β-oxidation, mitochondrial membrane transport, purine ribonucleotide metabolism, carbohydrate metabolism, and oxidative phosphorylation (FDR < 0.05; Figure 4C; Tables S1H and S1I).

Next we identified putative regulatory key driver genes in skeletal muscle samples obtained from the ExcHMDP and MyoGlu participants.^45,46^ Briefly, key driver analysis (KDA) identifies gene hubs by overlaying DEGs onto previously generated gene regulatory networks. KDA of muscle adaptation to exercise intervention identified *Myoz2* and *Esrrb* in the mouse, *SSC5D* and *SRPX2* in normal-weight men, and *APLN* and *ABLIM3* in overweight men (FDR < 0.05; Figure 4D). In both normal and overweight men, key driver gene networks were associated with inflammatory signaling and extracellular matrix, whereas mouse key driver gene networks were related to mitochondrial processes and muscle contraction.

### Molecular responses to exercise in adipose tissue

**Figure 5.**
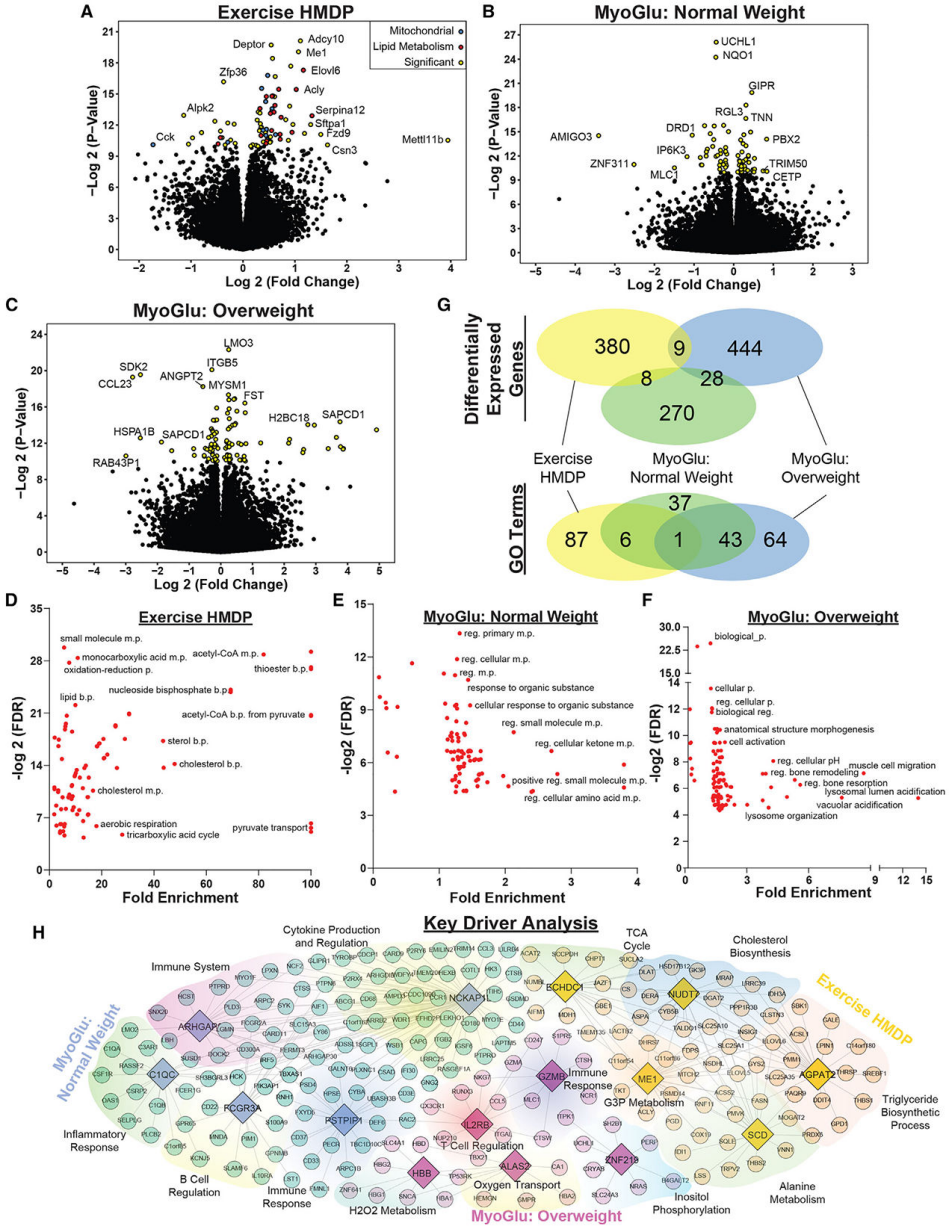
The impact of long-term exercise on white adipose tissue transcripts and key driver genes (A) Volcano plot of transcript expression in gonadal white adipose tissue across all female strains relative to the sedentary group. Significant genes (FDR < 0.05) are color coded with certain functional groups emphasized. (B and C) Volcano plot of gene expression from subcutaneous white adipose tissue in normal-weight and overweight humans relative to baseline values. Significant transcripts (FDR < 0.05) are color coded. (D–F) Gene enrichment analysis of DEGs from the (D) exercise HMDP, (E) MyoGlu normal weight, and (F) MyoGlu overweight. Only significantly enriched groups are displayed (FDR < 0.05). (G) Venn diagram showing the overlap among groups for DEG or GO terms. (H) KDA of DEGs colored by group. Key driver genes are enlarged in diamonds and the corresponding regulated genes within surrounding shaded regions. Top GO term for each shaded region is indicated.

Long-term exercise significantly reduced adipose tissue mass in humans and mice (Figures 2C, 3A, and 3B). To identify molecular transducers of this physiological adaptation to daily activity, we performed transcriptomics on gonadal white adipose tissue (gWAT) from the ExcHMDP and subcutaneous white adipose tissue (scWAT) from MyoGlu subjects. ExcHMDP gWAT gene expression was enriched for mitochondrial and lipid metabolism (FDR < 0.05; Figure 5A). Differentially expressed transcripts were associated with acetyl-CoA and pyruvate transport, cholesterol metabolism, tricarboxylic acid (TCA) cycle, and purine nucleoside metabolism (FDR < 0.05; Figure 5D). For overweight men, several lysosomal enrichment terms in addition to ERK1/2 signaling, blood vessel formation, and leukocyte activation emerged (FDR < 0.05; Figures 5C and 5F). Transcripts significantly changed by exercise intervention in normal-weight men were enriched for IL-1 signaling, amino acid, and ketone metabolism, as well as transcriptional regulation (FDR < 0.05; Figures 5B and 5E). Integrated analysis of transcript expression and enrichment between mice and humans revealed few DEG and Gene Ontology (GO) terms overlapping between species, a finding in contrast to skeletal muscle displaying high inter-species DEG and GO term concordance (Figure 5G; Table S1I). KDA was performed to identify potential regulatory transcripts for the exercise response in white adipose. Both normal-weight and overweight men displayed key drivers involved in immune and inflammatory responses, whereas mouse key drivers included cholesterol and triglyceride metabolism, and the TCA cycle (Figure 5H). We connected the exercise-induced changes in both mRNA expression and adipose tissue mass by performing candidate gene identification analysis. Seven transcripts were predicted to regulate adipose tissue weight loss during exercise intervention, including *Clic4, Frmd4a, H2-Ob, Mill1, Prxl2a, Snx9,* and *Tomm5* (FDR < 0.05; Figure S9A).

GWAS performed on gWAT from the ExcHMDP revealed differences in associated loci between groups, SED vs. TRN, suggesting different mechanisms of genetic regulation or adipose mass as a consequence of exercise training (Figure 2B). Next, GWAS was performed to interrogate the genetic architecture underlying the within-strain difference in gWAT mass between SED and TRN. No loci reached statistical significance (p > 4.1 × 10^−6^; Figure S9B). We then calculated a genome-wide polygenic score for each strain from this specific GWAS. Using ~400 SNPs, we identified a strong correlation between the genome-wide polygenic score and the difference in gWAT mass (p < 0.01, *R*^2^ = 0.52; Figure S9C). This relationship was consistent irrespective of the method employed for determination of genome-wide polygenic score. These findings suggest a strong interaction between genetics and exercise for adipose tissue weight loss during training intervention (Figure S9). Considering that the difference in adipose tissue mass following training (gWAT delta) did not correlate significantly with daily running distance in the ExcHMDP (p > 0.05, *R*^2^ = 0.04; Figure S9D), a further and more robust dissection of the interaction between genetics and exercise, as well as biological sex, in the control of adipose tissue weight loss is warranted.

### Molecular responses to exercise in liver

**Figure 6.**
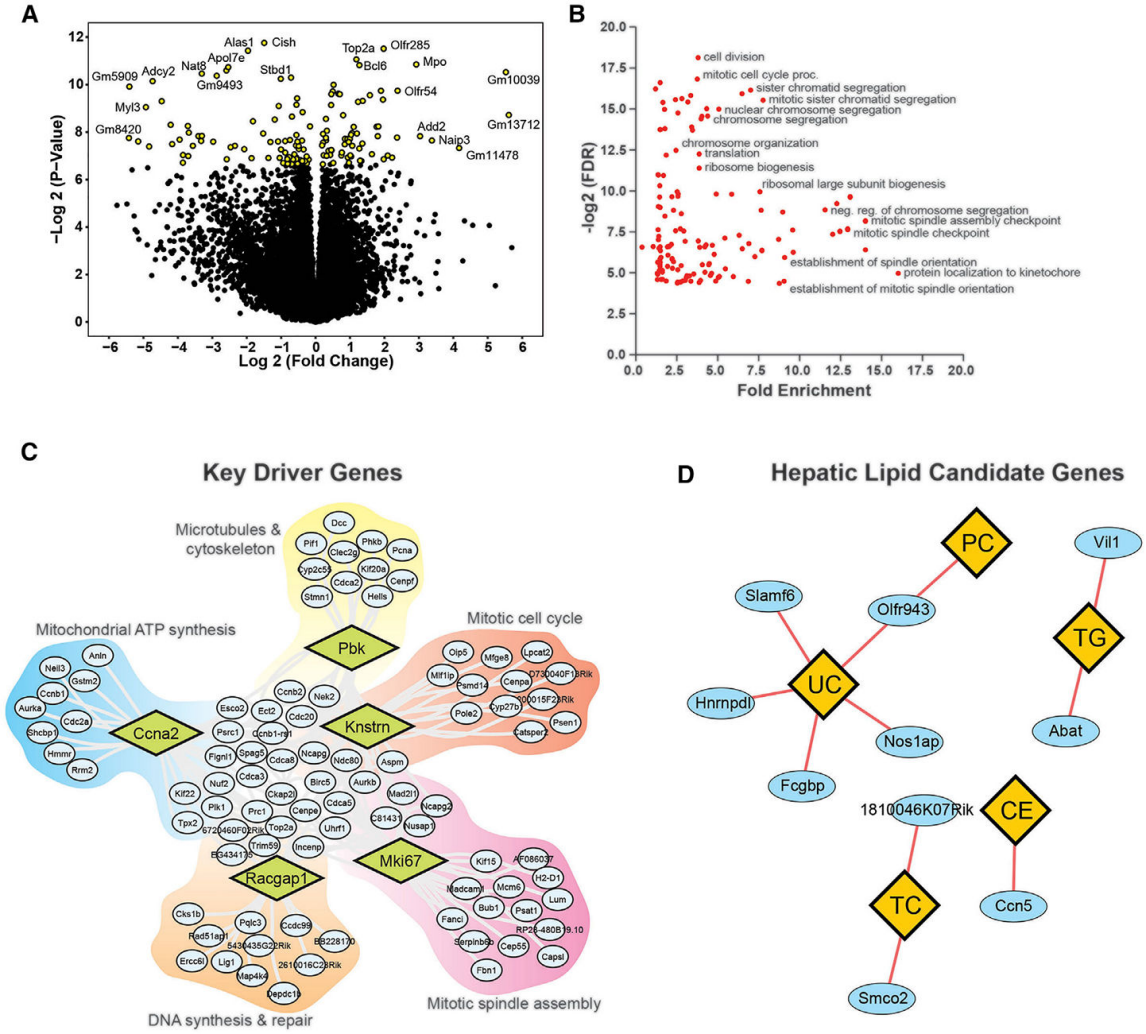
The impact of long-term exercise on liver transcripts and key driver genes (A) Volcano plot of hepatic transcripts across all strains following exercise training relative to sedentary. Significant transcripts (FDR < 0.05) are colored yellow. (B) Gene enrichment analysis of DEGs from liver samples of the ExcHMDP. Only significantly enriched groups are displayed (FDR < 0.05). (C) KDA of DEGs colored by group. Key driver genes are enlarged within diamonds and corresponding regulated genes are within surrounding shaded regions. Top GO term for each shaded region is indicated. (D) Candidate gene analysis (ovals) for liver lipid traits (diamonds).

The impact of long-term exercise on liver is understudied and less well appreciated compared with other metabolic tissues responsible for the mechanical work of physical activity.^47-49^ The ExcHMDP showed differences in hepatic physiological and molecular phenotypes (Figure 2C). Transcriptomic analysis of liver showed fewer DEGs compared with skeletal muscle and gWAT (FDR < 0.05; Figures 6A and S12A). Enrichment analysis of DEGs in female mouse liver following training were related to mitosis, cell division, cell cycle, and cytokinesis (FDR < 0.05; Figure 6B). Nearly 60% of all hepatic enrichment terms were associated with mitosis and cell cycle. KDA of the liver transcriptome consistently identified transcripts associated with mitotic processes such as *Mki67,* a known regulator of chromosomes during mitosis and a marker of cell proliferation (FDR < 0.05; Figure 6C). Candidate gene analysis for regulators of liver lipids and hallmarks of non-alcohol fatty liver disease revealed 10 gene candidates for the five liver lipids (p < 0.01; Figure 6D).

### Molecular response to exercise in heart

Exercise reproducibly improves cardiovascular function.^50,51^ Because heart weight was increased in TRN vs. SED animals (increased in 85% of HMDP strains; Figure 2C), we conducted cardiac transcriptomics. Similar to the liver, the heart showed relatively few DEGs compared with skeletal muscle and gWAT for SED vs. TRN mice (FDR < 0.05; Figure S11A). Enrichment analysis of DEGs displayed several inflammatory and immune processes (leukocyte regulation, macrophage activation, and *TNFα* and other cytokine products), calcium signaling and regulation, muscle growth and development, and angiogenesis (FDR < 0.05; Figure S11B). These biological processes overlap with those identified in skeletal muscle. Candidate gene identification analyses of exercise-induced cardiac hypertrophy identified five potential regulatory transcripts: *IL31ra, Fam167b, Tafa5, Crip3,* and *Nanos1* (p < 0.01; Figure S11C).

### Molecular responses to exercise in brown adipose tissue

Brown adipose tissue (BAT) has received increasing attention in the literature, especially with respect to its role in energy expenditure.^52-54^ We observed a small but significant increase in BAT mass following training in the ExcHMDP (p < 0.05; Figure 2C). The increase in BAT mass prompted us to investigate the effect of exercise on the BAT transcriptome. Unexpectedly, BAT had the most DEG of all tissues assessed (FDR < 0.05; Figures S12A and S13A). Downregulated BAT transcripts following exercise training were significantly enriched for several RNA regulatory processes, including poly(A) tail shortening, splicing, RNA polymerase II transcription, and histone H4 acetylation (FDR < 0.05; Figure S12B). BAT transcripts upregulated following exercise training were significantly enriched for metabolic processes including pyruvate metabolism, the mevalonate pathway, glycolysis, pentose phosphate pathway, cholesterol biosynthesis, gluconeogenesis, mitochondrial biogenesis, fatty acid metabolism, and glycogen metabolism (FDR < 0.05; Figure S12C). Considering the noted species and biological sex differences described in the literature regarding the role of BAT in the regulation of energy expenditure and its involvement in metabolic adaptation to exercise training, additional comparative studies are needed.^55^

### Integrated analysis and Web application

A primary goal of our research was to improve understanding of the integrated physiological responses to exercise. Thus, we compared DEGs and GO enrichment categories between tissues within the ExcHMDP. Skeletal muscle, and brown and white adipose tissue were most similar (Figures S13A and S13B). Although heart and liver were different from all other tissues. Only four transcripts (*Slc25a1, Acly, Ccn1,* and *Dusp1*) were differentially expressed between SED and TRN in all tissues except liver (Table S1M).

We reasoned that exercise likely elicits harmonized tissue responses throughout the body to coordinate metabolic responsiveness. Thus, it is possible that gene programs are synchronized between tissues during exercise by changes in cell communication. To gain insight into coordinated tissue responses, we interrogated inter- and intra-tissue relationships between gene modules (p < 0.01; Figure S13C). We identified 20 modules that possessed at least one significant inter-tissue correlation. Furthermore, we identified certain modules residing at the nexus of multiple inter-tissue correlations. The presence of inter-tissue module connections suggests a secreted factor-mediated pattern of communication, a topic currently under investigation by our research team.

Similar to our analysis of physiological traits (Figure S5A), we performed an unbiased PCA using the transcriptomes of five tissues from the ExcHMDP (Figure S13D). These analyses showed clustering by strain rather than group (SED vs. TRN). However, when interrogating the top 500 DEGs from each tissue (the average number of DEGs in a tissue), a significant separation by group (exercised vs. sedentary) was observed (p < 0.001; Figure S13E). These findings reflect the existence of a conserved exercise program masked by exercise unresponsive strain- and tissue-dependent transcripts.

**Figure 7.**
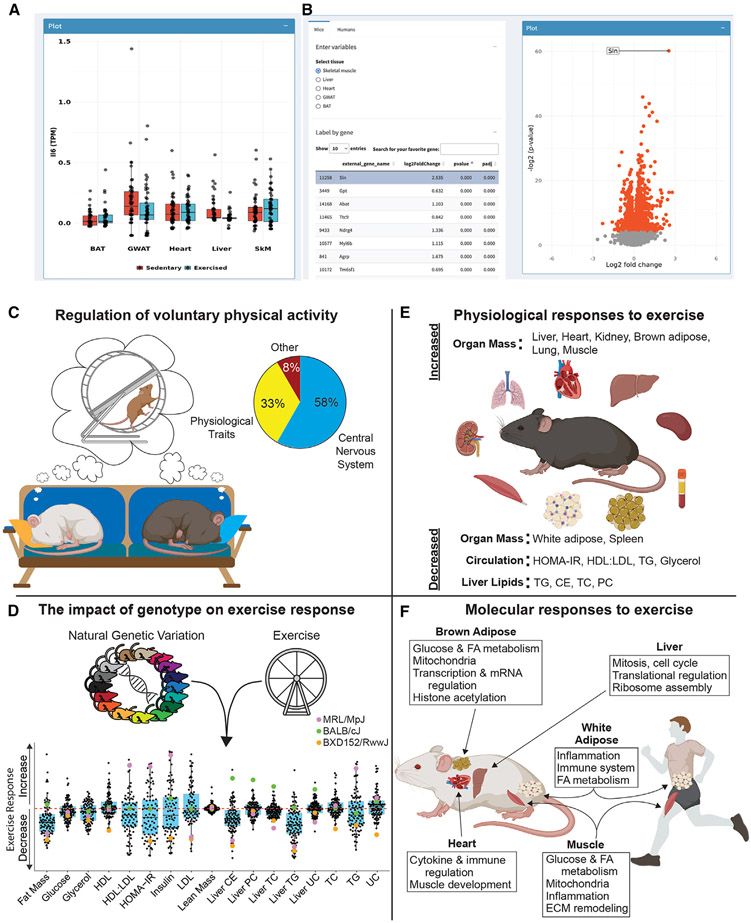
Overview of Shiny Web application and summary of findings. (A) Screen shot of the Web application showing dot plots of transcriptomics data from BAT, gWAT, heart, liver, and skeletal muscle (SkM) of sedentary vs. exercised (trained) mice. (B) Screen shot of time variable comparisons for human muscle transcriptomic findings in table format (left) and volcano plot (right). The website enables users to browse, mine, as well as download transcriptomics data from mice and humans. (C–F) Summary of the main findings divided into four broad categories: (C–F) volitional exercise, (D) genotype-specific exercise response, (E) physiological responses to exercise intervention, and (F) molecular adaptations to repeated physical activity.

The overarching goal of the ExcHMDP was to develop an expandable, user-friendly, and open-access resource for the scientific community. All transcriptomic data from mice and visual representation of tissue transcripts from humans are publicly available at https://exchmdpmg.medsch.ucla.edu/app/. This Web site application enables side-by-side comparisons of expression and DEGs between tissues, species, exercise groups, and exercise time points (Figures 7A and 7B) and was designed for ease of data mining to advance hypothesis generation by the research community.

## DISCUSSION

Combining the power of genetics, multi-omics, deep phenotyping, and data integration, we provide species-specific, as well as species-conserved, pathways associated with exercise adaptations including (1) phenotypic responses to exercise for physiologically relevant traits; (2) tissue-specific molecular responses to exercise in skeletal muscle, white adipose tissue, BAT, liver, and heart; as well as (3) adaptations as a consequence of interactions between exercise workload and genetic variation (Figure 7C). The integration of phenotypic and molecular data identified regulatory genes for whole-organism and tissue-specific phenotypic effects of exercise (e.g., adipose tissue mass reduction). Bioinformatic analyses led to the identification of putative regulators of voluntary physical activity, most strongly controlled by the hypothalamus. Finally, data have been made publicly available in a Web-based application allowing for hypothesis development and exploration (https://exchmdpmg.medsch.ucla.edu; Figures 7A and 7B). This Web application provides opportunity for users to compare different datasets between species, tissues, exercise groups, and exercise time points. This Web application is expandable and will be utilized as a study repository as additional HMDP data become available.

The major goal of this research was to improve understanding of the effects of genetic architecture on metabolic tissue adaptation to long-term physical activity. Our findings in mouse and humans substantiate known physiological outcomes of exercise (e.g., reductions in adipose mass and circulating lipids, and increases in lean mass and insulin sensitivity).^56-60^ We also provide evidence supporting the impact of exercise on relatively understudied tissues and traits (e.g., liver, spleen, and kidney adaptations). Genetic investigation of metabolic traits revealed that most QTLs were not shared between TRN and SED. This suggests exercise-specific regulatory mechanisms for phenotypic traits and a transition of physiological status from sedentary to exercise trained. Group-specific QTLs have been observed in previous HMDP studies where interventions to induce trait outcomes reflective of disease pathobiology were studied.^37^ Moreover, in translation to humans, exercise-specific QTLs may provide a conserved resilience when non-specific stress is imposed compared with untrained individuals, as previously proposed.^61,62^

In addition to group outcomes, another major finding of our work relates to individual phenotypic responses to exercise in both humans and mice (Figures 7C, S5A, S5B, S13D, and S13E). For example, strains MRL/MpJ, BALB/cJ, and BXD152/RwwJ were similar in body mass and running wheel distance, speed, and duration. However, in contrast to BALB/cJ and MRL/MpJ strains, in which exercise contributed minimally to metabolic health outcomes, BXD152/RwwJ mice improved markedly in the aggregate metabolic health index following training. In the MyoGlu trial, where exercise intervention was tightly controlled, one normal-weight individual experienced a reduction in insulin sensitivity (assessed by the gold-standard method hyperinsulinemic-euglycemic clamp) and liver fat, whereas a second normal-weight individual showed improvement in insulin sensitivity. Although the existence of a true exercise non-responder is contended,^63^ our findings reflect a differential effect of exercise on metabolic health underpinned by genotype.^28^ Our findings corroborate similar observations of prior gene-environment interaction studies.^17,45^ This point is further emphasized by examining two genetically similar strains, BXD56/RwwJ and BXD102/RwwJ, where both strains exhibited similar beneficial effects of exercise training, despite running 0.9 and 11.6 km per night, respectively. How physical workload affects the multi-ome to produce unique signaling and communication nodes requires further dissection. In an effort to advance precision medicine, methodologies to predict individual metabolic responsiveness to exercise or development of algorithms to derive a genome-wide polygenic score will advance the discipline of personalized exercise prescription.

Transcriptomic profiling of the liver following long-term exercise revealed that ~60% of GO terms derived from differentially expressed transcripts were associated with mitosis and the cell cycle. Moreover, KDA of liver transcriptomics identified hub genes involved in the cell cycle. Two hepatic modules enriched for cell cycle and mitosis genes were identified. Liver mass was significantly increased in TRN vs. SED animals despite reductions in lipid content.^64^ Although the increase in tissue weight following exercise was initially thought to be a consequence of hepatic glycogen supercompensation, as previously described,^65,66^ our findings suggest liver hyperplasia. Although we cannot rule out an increase in non-parenchymal cells following training, our observation of an increase in traditionally quiescent hepatocytes post exercise training requires further interrogation. One potential explanation could be a physiological adaptation by the liver to meet the increased metabolic demand of physical activity. Considering recent findings showing that exercise prevents and or delays hepatocarcinogenesis independent of weight loss, our observations may be of important clinical relevance.^67,68^

We utilized the ExcHMDP to better understand how genetics control transcriptome remodeling in BAT following long-term exercise. Although there are conflicting findings with respect to exercise adaptation in beige adipose tissue and BAT, we found that mouse BAT, over all other tissues studied, was increased in mass and displayed the greatest number of DEGs in response to exercise intervention. Considering the evidence that exercise activates BAT in rodents,^52,53^ but not humans,^54,55^ a more nuanced understanding of species differences as well as responsiveness to environmental cues (e.g., ambient temperature and diet) requires greater consideration.^52-54,69,70^ Our transcriptomic analysis of BAT suggests an increase in glucose and fatty acid metabolism with concomitant downregulation of genes associated with transcription suppression. Our findings support that continued research investigating the role of BAT during exercise, as well as its contribution to systemic metabolism and disease prevention, is warranted.

Previous studies have interrogated factors driving volitional physical activity in both mice and humans with emphasis on the genetic architecture underlying this trait.^25,26,71-73^ Advanced intercross mice and human epidemiological observations revealed a moderate to high heritability for physical activity and dozens of QTLs. QTL mapping revealed five significant and eight suggestive QTLs for body weight (Chr 4, 7.54 Mb; confidence interval [CI] 3.32–10.34 Mb; Bwq14), body composition, wheel running duration (Chr 16, 33.2 Mb; CI 32.5–38.3 Mb), body weight change in response to exercise (1: Chr 6, 77.7Mb; CI 72.2–83.4 Mb and 2: Chr 6, 42.8 Mb; CI 39.4–48.1 Mb), and food intake during exercise (Chr 12, 85.1 Mb; CI 82.9–89.0 Mb). The intrinsic motivation to participate in leisure time physical activity is driven by an interaction between genetic, environmental, and socioeconomic factors.^74^ Our findings confirm a 68% heritability for daily running distance in female mice, one of the highest heritability values of all traits assessed in the ExcHMDP. We integrated these data with previous HMDPs and identified a strong connection between daily running distance and gene expression in specific regions of the CNS, specifically the hypothalamus. The connection between daily running distance and the CNS transcriptome was more significant than its connection to the cardiovascular system, lean muscle mass, or specific circulating hormones and metabolites. The hypothalamus has previously been associated with volitional physical activity in both humans and rodents.^75,76^ Our bioinformatic analyses identified 81 candidate genes in hypothalamus associated with daily running distance. Although the hypothalamus may be a central regulator of daily running distance in the mouse, our findings suggest additional inputs from other brain regions, including the hippocampus and striatum, as well as peripheral tissues. Continued investigation into the central and integrative signals from the periphery that drive voluntary physical activity and improved strategies promoting lifelong exercise prescription adherence are warranted.

In conclusion, this research makes publicly available a longitudinal, cross-species, and integrated analysis of adaptations to acute and long-term exercise intervention with specific emphasis on genetic regulators of metabolic health. Importantly, we showed that there are exercise-conserved gene signatures and metabolic trait adaptations from mouse to human. Moreover, certain metabolic traits are highly influenced by genetic architecture, and thus, despite performance of a matched exercise workload, trait adaptation can be genotype specific. Thus, identification of genetic drivers underlying metabolic health adaptation to exercise intervention in diverse human populations is critical. The overarching goal is that the vast data repository we generated will serve as a resource to be leveraged for target validation and novel hypothesis generation as well as to drive personalized exercise prescription to patently reduce metabolic disease burden.

### Limitations of the study

Limitations of our work should be considered when interpreting our findings, and these are primarily related to the scale of the ExcHMDP project. Specifically, because we studied 100 mouse strains over numerous months, we were unable to ascertain and synchronize mouse estrous cycles. Our position is that the inherent genetic differences between strains and the metabolic effects of exercise training far outweigh the impact of estradiol cyclicity on complex trait outcomes observed for the 100-strain mouse panel. To understand volitional exercise drive, the mouse panel performed wheel running; however, not all strains performed equal volumes of exercise. Many prior rodent studies controlled workload by using a treadmill and forced activity typically motivated by a stressful stimulus (e.g., electric shock). Differences in exercise modality and stress response to activity will likely preclude inter-study comparisons with the ExcHMDP, as marked differences in hormone and tissue responses associated with forced vs. volitional activity are known.^77^ Forced running during early adolescence in well-studied Sprague-Dawley rats showed a sexual dimorphism in weight and body volume as well as relative adipose tissue mass following training.^78^ These findings in female rats in the context of forced wheel running are not supported by our observations in mice engaging in volitional activity. Published work suggests that sex-dependent effects of exercise on body composition might vary depending on animal age, strain,^79^ and exercise mode (i.e., voluntary vs. forced).^4,80,81^ We intend to interrogate differences between volitional and forced activity in rats and mice in addition to the time course of transcript alteration in the post-acute and chronic exercise-trained conditions in collaboration with the MoTrPAC consortium. The primary advantage of our work using the HMDP is that we can compare gene-gene and gene-trait relationships across a variety of mouse panel studies to contrast the effects of exercise with dietary or drug intervention by sex.

Because most metabolic tissues include a heterogeneous mix of cell types, and because exercise alters the cell composition of most tissues, our bulk RNA sequencing approach limits our insight of exercise-induced adaptation in cell composition within tissues.^82,83^ Findings from single-cell and single-nuclei sequencing, as well as spatial transcriptomic studies, will help resolve questions related to cell composition within the transcriptional landscape of a tissue.

Last, translational relevance is always a concern when comparing rodents with humans. We compared men with female mice employing different modes of exercise, which presumably affected trait outcomes and reduced the number of DEGs overlapping between species after training. In light of these limitations, we consider the differentially expressed transcripts that were identified between SED vs. TRN mice and humans to be robust, conserved, and selectively exercise-responsive transcripts independent of sex, species, and mode of activity. In ongoing studies, we are more rigorously exploring the impact of sex on the transcriptomic response to exercise within and between species. It is likely that our current work missed sex-specific adaptations that may ultimately lead to new hypotheses about drivers of volitional activity as well as hormone- and sex chromosome-specific drivers of exercise-induced health benefit.

## STAR★METHODS

### RESOURCE AVAILABILITY

#### Lead contact

Additional information and requests for resources and reagents should be directed to Andrea L. Hevener (ahevener@mednet.ucla.edu).

#### Materials availability

This study did not generate new reagents.

#### Data and code availability

- RNAsequencing data can be found at https://exchmdpmg.medsch.ucla.edu/app/ as well as online data repositories ExcHMDP data GEO:GSE230102, GSE64770, GSE16780, GSE121098, and MyoGlu data GSE227419.
- The original code has been deposited at Zenodo is publicly available as of the date of publication. DOI is listed in the key resources table.
- Any additional information required to reanalyze the data reported in this paper is available from the lead contact upon request.

### EXPERIMENTAL MODEL AND SUBJECT DETAILS

#### Human subjects MyoGlu

Briefly, the MyoGlu study^18^ included healthy sedentary (<1 bout of exercise per week the previous year) men (40–65 years) divided into two groups stratified by BMI: overweight (BMI 29.5 ± 2.3 kg/m^2^) or normal weight controls (BMI 23.6 ± 2.0 kg/m^2^). Both groups (n = 26) underwent combined strength and endurance training for 12 weeks, including two endurance bicycle sessions (60 min) and two whole body strength training sessions (60 min) per week. A 45 min bicycle test at 70% of VO_2_ max was performed before and after the 12-week intervention period. Skeletal muscle (*vastus lateralis*) and subcutaneous white adipose tissue biopsies were taken before and after a 12-week of exercise intervention, 48h after the last bout of activity. Assessment time points included: baseline at rest, baseline immediately after a 45 min exercise session, 2 h after the 45 min exercise session, at rest 12 weeks after exercise intervention, immediately following another 45 min exercise session 12 weeks after exercise intervention, and 2 h after the 45 min exercise session 12 weeks after exercise intervention. MyoGlu was a controlled clinical trial (clinicaltrials.gov: NCT01803568) and adhered to the Declaration of Helsinki. The National Regional Committee for Medical and Health Research Ethics North, Tromso, Norway approved the study, with reference number: 2011/882. Written informed consent was obtained from all participants before any study-related procedure.

#### Mouse strains

All studies were approved by the Institutional Animal Care and Use Committee (IACUC) and the Animal Research Committee (ARC) at the University of California, Los Angeles (UCLA). Female mouse strains of the ExcHMDP are listed in Table S1A and were acquired from The Jackson Laboratories (Bar Harbor, ME, USA) or through Dr. Rob Williams at the University of Tennessee Health Science Center at 10 weeks of age. Mice were maintained on a strict 12-hr light/dark cycle (6am to 6pm) with *ad libitum* access to standard rodent chow (Teklad 8604, Envigo, Indianapolis, IN, USA) and water. Sedentary mice were housed 1-4 animals per cage. Exercised mice were individually housed with continuous access to an in cage running wheel monitored by VitalView^®^ Activity Software (Starr Life Sciences, Oakmont, PA, USA) for 30 days beginning training at 12 weeks of age. After 30 days, running wheels were locked between 6-9 am local time. 24h post exercise cessation, cages were replaced and chow removed from all animals 6h prior to euthanasia. Animals were euthanized between 12-4 pm local time. Samples were removed in the following order: blood from the abdominal aorta, gonadal white adipose tissue, quadriceps, inguinal white adipose tissue, heart, lungs, liver, spleen, kidney, colonic feces, hindlimb (gastrocnemius-plantaris-soleus), and brown adipose tissue. Whole blood was deposited into K3 EDTA-coated tubes and centrifuged for five minutes at 3000 G with plasma collected on ice. All samples excluding plasma and colonic feces were quickly rinsed in sterile saline, pat-dried, weighed, and frozen in liquid nitrogen. All samples were stored at −80°C for subsequent analysis.

Daily running distance was calculated as the average running distance per day over the experiment timeframe. Average running speed was calculated by normalizing all 15 second intervals with values > 0 relative to 1 second. Percent of time running was calculated by dividing the sum of 15 second intervals > 0 by the sum of all 15 second intervals.

### METHOD DETAILS

#### Plasma hormone and metabolite analyses

##### Liver and plasma metabolite analyses

Plasma metabolites and HOMA-IR^17^ for humans and mice as well as liver lipids for mice only were analyzed^45^ using commercially available kits as per manufacturer instructions.

#### Euglycemic-hyperinsulinemic clamp studies

Euglycemic-hyperinsulinemic clamp studies were performed after an overnight fast. A fixed dose insulin (40 mU/m^2^ x min^−1^) was infused, and glucose infusion (200 mg/mL) was adjusted to maintain euglycemia (5.0 mmol/L for 150 min).^18^ Insulin sensitivity is reported as glucose infusion rate (GIR; mg x kg^−1^ x min^−1^) during the last 30 min of the clamp. Whole blood glucose was measured by glucose oxidase method (YSI 2300, Yellow Springs, OH), and plasma glucose was calculated (whole blood glucose x 1.119).

#### Tissue trait analyses

##### Trait by trait correlations

Biweight midcorrelation was calculated for pairwise trait correlations within each group using the WGCNA package in R. The sedentary and exercised trait correlation matrix was visualized using the ‘ComplexHeatmap’ package^84^ in R. For trait by trait correlations involving a group difference (sedentary subtracted from trained value for each strain, or trait delta), a random pairing method was used. Briefly, for each strain, a sedentary mouse was randomly chosen and the value for that trait was subtracted from a randomly chosen trained mouse. This process was continued without replacement for each group until all mice were utilized. If the sedentary group number was greater than the trained group number, a randomly chosen trained mouse was used twice. If the sedentary group number was less than the trained group number, a randomly chosen sedentary mouse was used twice. This enabled all mice within a strain to be included. Where strain averages for traits were employed, this process was repeated 1000 times with the results from all trials averaged to give a final group difference for each strain.

#### Heart phenotype correlations

Heart phenotypes from a previous sedentary, untreated HMDP (age matched)^37^ were correlated with running distance per day, as well as trait-by-trait comparisons between sedentary vs. trained mice of the ExcHMDP.

#### Cumulative exercise effect: Metabolic traits

The cumulative exercise effect on metabolic traits was calculated as follows using traits where the general exercise effect is known.^85^ For the ExcHMDP, the traits included muscle mass, fat mass, liver triglycerides, and plasma insulin, triglyceride, glucose, and HDL. Heart, quadriceps, and gastrocnemius mass were added to comprise muscle mass. Gonadal white adipose tissue and inguinal white adipose tissue were added to comprise fat mass. The strain average was determined for each trait within a group. Next, the strain percent change was determined for each trait. All traits were then summed giving a single final value for each strain indicated as the cumulative exercise effect. For MyoGlu, the same process was performed. Traits included fat free mass, fat mass, liver fat, glucose infusion rate (GIR), HDL, and plasma triglyceride (TG).

#### Single cell and bulk RNAsequencing and data processing

##### RNA Isolation, library preparation, and sequencing

Whole quadriceps, gonadal white adipose tissue, heart, brown adipose tissue, and a portion of the liver were pulverized at the temperature of liquid nitrogen. Tissue was homogenized in Trizol (Invitrogen, Carlsbad, CA, USA), RNA was isolated using the RNeasy Isolation Kit (Qiagen, Hilden, Germany), and RNA concentration and quality were assessed (RIN >7.0 used in downstream applications). Libraries were prepared using KAPA mRNA HyperPrep Kits and KAPA Dual Index Adapters (Roche, Basel, Switzerland) per manufacturer’s instructions. A total of 800-1000 ng of RNA was used for library preparation with settings 200-300 bp and 12 PCR cycles. The resultant libraries were tested for quality. Individual libraries were pooled and sequenced using a HiSeq 3000 or NovaSeq 6000 S4 following in house, well established protocols by the UCLA Technology Center for Genomics and Bioinformatics (TCGB).

#### Single cell RNA sequencing

Six female C57BL/6J mice were subjected to the same exercise protocol as ExcHMDP animals. Following animal euthanasia, the hypothalamus from 3 sedentary and 3 exercise trained mice underwent a DropSeq single cell protocol.^86^ The resulting gene matrices for each sample were combined yielding two groups and these were further analyzed using Seurat v2.3.4.^87^ The mouse brain atlas was used to annotate cells within each cluster.^88^ Two marker genes were used to identify each cluster.

#### Heritability

SNP-heritability was calculated for mice of the ExcHMDP.^15^

##### Genome wide association analyses

Genome wide association analyses were conducted.^15^ Quantitative trait loci (QTLs) were considered distinct between groups if the significant locus was more than 20 Mb from a locus in the other group and below the suggestive significance threshold (p < 4.1x10^−5^).

##### Candidate gene identification

Candidate genes in GWAS loci were prioritized based on known biologic function or correlation in co-expression with a specific trait. In particular, genes whose *cis*-regulation was correlated with the trait were considered as highly likely candidate genes.^89^ Briefly, when only exercise trained animals were used, SNPs within 1 Mb (*cis*-acting) of a gene with a *cis*-eQTL (*P* < 1E^−4^) were identified. The median of the allele-specific expression for each SNP of that gene was calculated and those values were then correlated with a particular trait. For candidate genes identified using both sedentary and exercised animals, sedentary gene expression was subtracted from trained gene expression giving the exercise-induced change in gene expression. The trait in question underwent the same analysis where the sedentary value was subtracted from the trained value. Resulting values from the gene expression and the trait were then used as described above in downstream applications.

##### Genome wide polygenic score

Genome wide association analyses were conducted.^15^ Quantitative trait loci (QTLs) were considered distinct between groups if the significant locus was more than 20 Mb from a locus in the other group and below the suggestive significance threshold (p < 4.1x10^−5^).

#### Heritability

SNP-heritability was calculated for mice of the ExcHMDP.^15^

### QUANTIFICATION AND STATISTICAL ANALYSES

#### Statistical analysis of the biological data

Phenotype-phenotype correlations^15^ and principal component analyses were performed using the FactoMineR v2.3 and factoextra v1.0.7 packages in R.^90^ Groups differences were determined using the Vegan v2.5-6 package in R. Key driver analyses were conducted^45,46^ and the final resulting networks were visualized using Cytoscape 3.8.0.^91^

For the ExcHMDP and MyoGlu, raw RNAseq reads were inspected for quality using FastQC v0.11.9 (Barbraham Institute, Barbraham, England). Reads were aligned and counted using kallisto v0.45^92^ against the Ensembl mouse transcriptome (v97) to obtain counts and transcripts per million (TPM). Samples were analyzed for differential expression using DeSeq2 v1.28.0^93^ and were corrected using limma v3.44.1^94^ accounting for library prep batch and sequencing flow cell lane.

Gene enrichment analysis was conducted using Pantherdb (http://pantherdb.org/). Afalse discovery rate (FDR) < 0.05 was considered significant. Unless otherwise noted, values presented are expressed as means ± SEM. The two-sample Student’s t-test was used to examine the difference between the two groups. All analyses were performed using R v4.0.0, and p values <0.05 were considered statistically significant unless specifically stated. Figures were compiled and made using Graphpad Prism v9 (San Diego, CA, USA) or Adobe Illustrator v24.3 (San Jose, CA, USA).

### ADDITIONAL RESOURCES

A publicly available interactive web browser for tissue gene expression exploration of MyoGlu and ExcHMDP datasets analyzed in this study was created for hypothesis generation. Description: https://exchmdpmg.medsch.ucla.edu/app/. MyoGlu was a controlled clinical trial (clinicaltrials.gov: NCT01803568) and the ethical committee statement can be found at https://link.springer.com/article/10.1007/s00125-020-05296-0.

## Supplementary Material

Supplement Figures

Supplement Tables

## Figures and Tables

**Table:** KEY RESOURCES TABLE

REAGENT or RESOURCE	SOURCE	IDENTIFIER
Chemicals, peptides, and recombinant proteins		
QIAzol Lysis Reagent	Qiagen	79306
Chloroform, HPLC Grade	Thermo Fisher	C606-4
Isopropanol, 99.5%	Acros Organics	32727-0010
Critical commercial assays		
Cholesterol	In house	
Insulin ELISA	Alpco	80-INSMSU-E01
Glucose	StanBio Laboratory	1071-250
Triglyceride	Sigma Aldrich	TR0100
Phospholipid-C kit	WAKO Diagnostics	997-01801
Glycerol	Sigma Aldrich	FG0100
Qiagen	RNAeasy Kit	74106
Deposited data		
HMDP (raw and processed data)	Bennett et al.^14^ Parks et al.^17^	GSE64770
HMDP RNA expression profiling (multitissue studies, raw and processed data)	Norheim et al.^15^ Chella Krishnan et al.^45^	GSE16780 GSE121098
HMDP RNAsequencing (hypothalamic studies)	Hasin-Brumshtein et al.^40^	GSE79551
RNAsequencing MyoGlu Human Exercise Studies	Langleite et al.^18^	GSE227419
RNAsequencing Exc HMDP	This paper	GSE230102
Experimental models: Organisms/strains		
100 strain mouse panel	The Jackson Laboratory	Strain IDs in Table S1A
Software and algorithms		
Prism v9	GraphPad Software	https:www.graphpad.com
R Studio Posit Rv4.0.0	R Studio Desktop Complex Heatmap package	https://posit.com
WCGNA	This paper	https:cran.r-project.org
DESeq2	This paper	https://bioconductor.org/packages/release.bioc/html/DESeq2.html
Gene Enrichment Analysis	This paper	http://pantherdb.org
Graphics	This paper Adobe Illustrator v24.3	http://www.adobe.com
Graphics	This paper Biorender	www.biorender.com
Other		
Resource Website	This paper	https://exchmdpmg.medsch.ucla.edu/app
Code	This paper	https://github.com/tho-ols/exchmdpmg https://doi.org/10.5281/zenodo.7729799
Clinical Trial Registry - MyoGlu	Langleite et al.^18^	NCT01803568
HMDP RNAsequencing (hypothalamic studies)	Dryad Digital Repository	https://doi.org/10.5061/dryad.vm525

## Highlights

- Complex traits and tissue transcriptomics in men and 100-strain mouse panel
- Candidate gene analyses for genetic drivers of exercise-modified traits
- Non-overlapping quantitative trait loci for select traits of sedentary versus trained
- Publicly available data mining resource by user-friendly, Web-based application

## INCLUSION AND DIVERSITY

We support inclusive, diverse, and equitable conduct of research.
