# Supplementary material for: Conserved multi-tissue transcriptomic adaptations to exercise training in humans and mice
Source: Cell Rep. Author manuscript; Available in PMC 2024 Aug 28. (PMC11352395; doi:10.1016/j.celrep.2023.112499)
Supplement: Supplement Figures [file NIHMS2016076-supplement-Supplement_Figures.pdf]

Cell Reports, Volume *42*

Supplemental information

Conserved multi-tissue transcriptomic adaptations to exercise training in humans and mice

Timothy M. Moore, Sindre Lee, Thomas Olsen, Marco Morselli, Alexander R. Strumwasser, Amanda J. Lin, Zhenqi Zhou, Aaron Abrishami, Steven M. Garcia, Jennifer Bribiesca, Kevin Cory, Kate Whitney, Theodore Ho, Timothy Ho, Joseph L. Lee, Daniel H. Rucker, Christina Q.A. Nguyen, Akshay T.S. Anand, Aidan Yackly, Lorna Q. Mendoza, Brayden K. Leyva, Claudia Aliman, Daniel J. Artiga, Yonghong Meng, Sarada Charugundla, Calvin Pan, Vida Jedian, Marcus M. Seldin, In Sook Ahn, Graciel Diamante, Montgomery Blencowe, Xia Yang, Etienne Mouisel, Matteo Pellegrini, Lorraine P. Turcotte, Kåre I. Birkeland, Frode Norheim, Christian A. Drevon, Aldons J. Lusis, and Andrea L. Hevener

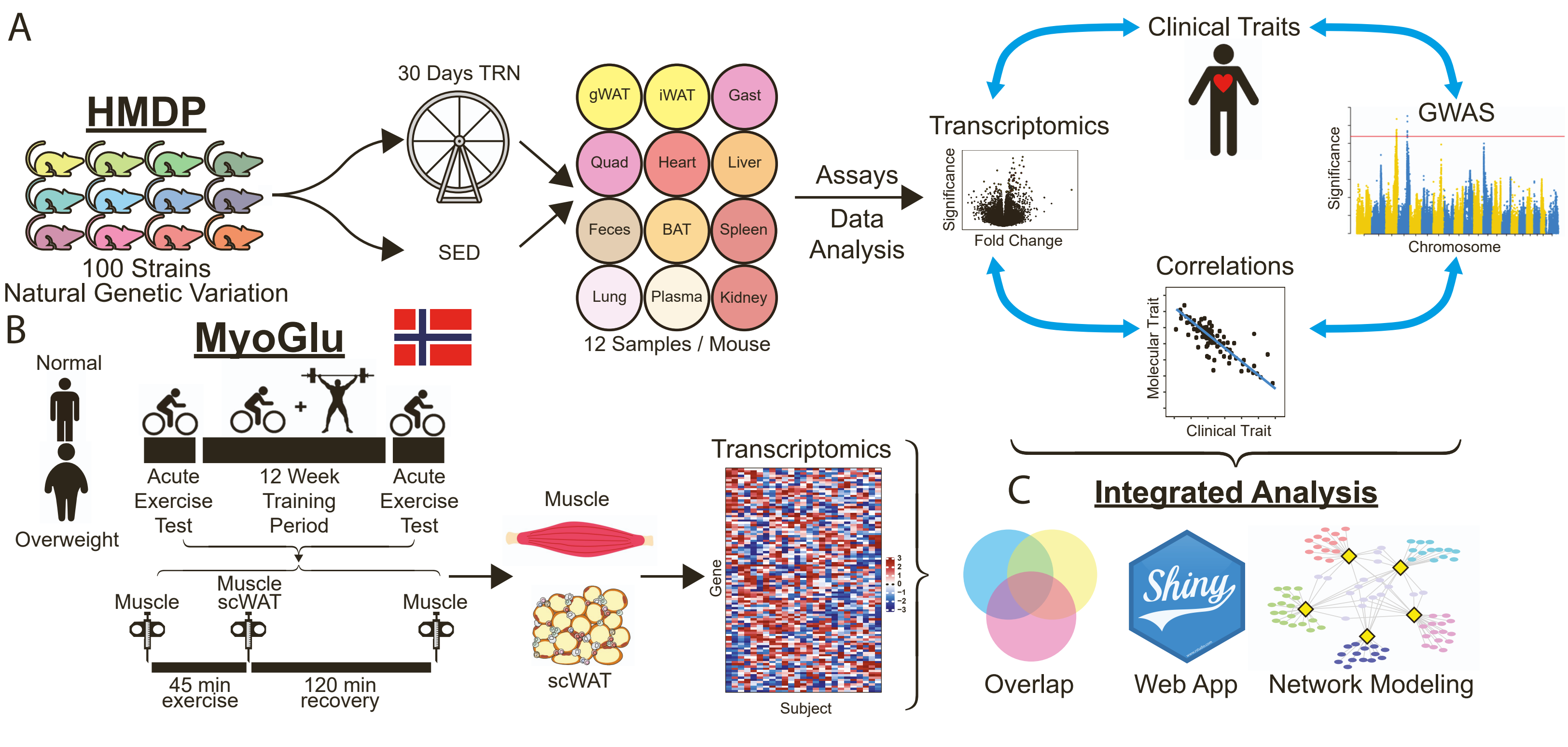

https://exchmdpmg.medsch.ucla.edu

Moore et al, Supplemental Figure 1

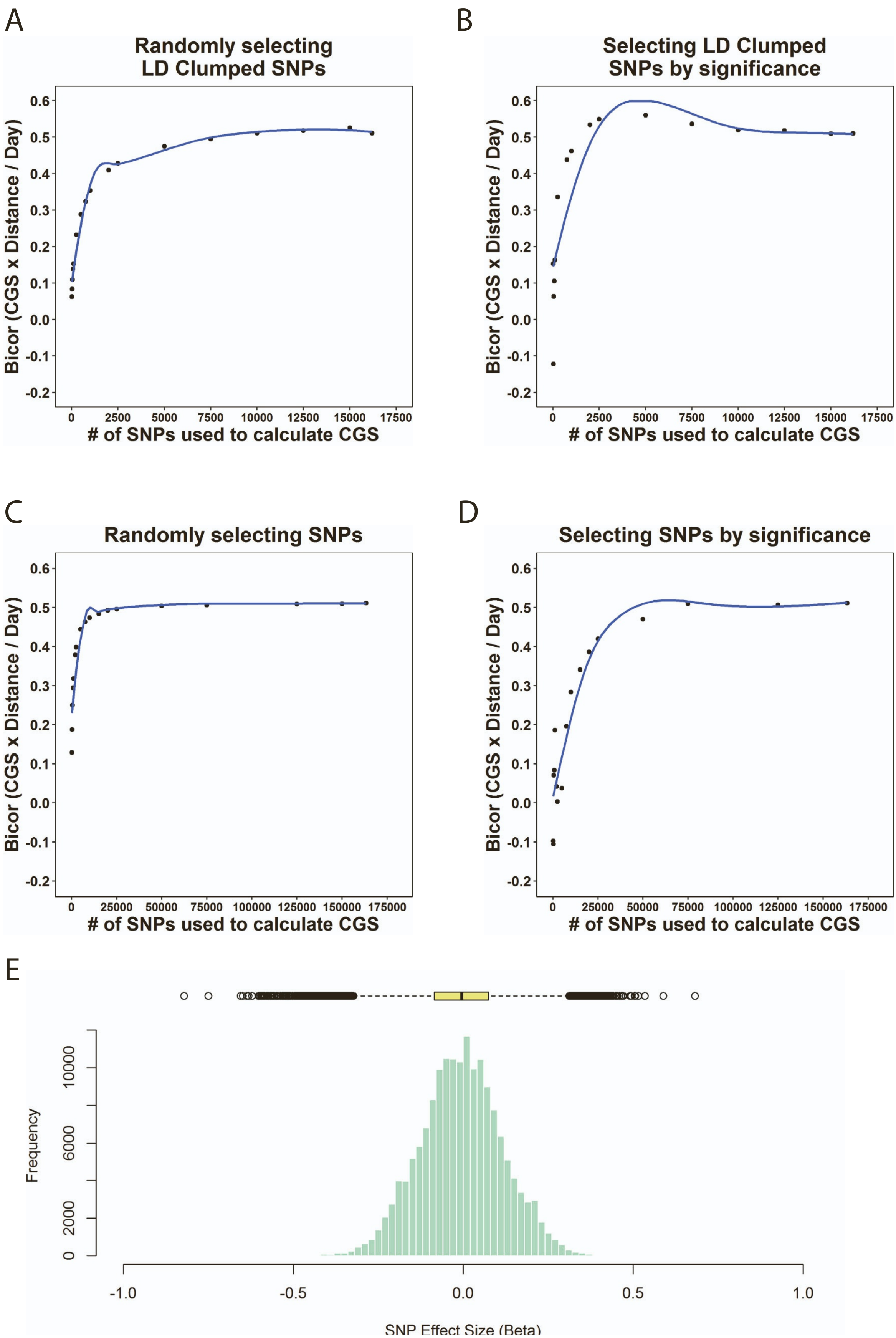

Moore et al, Supplemental Figure 2

A

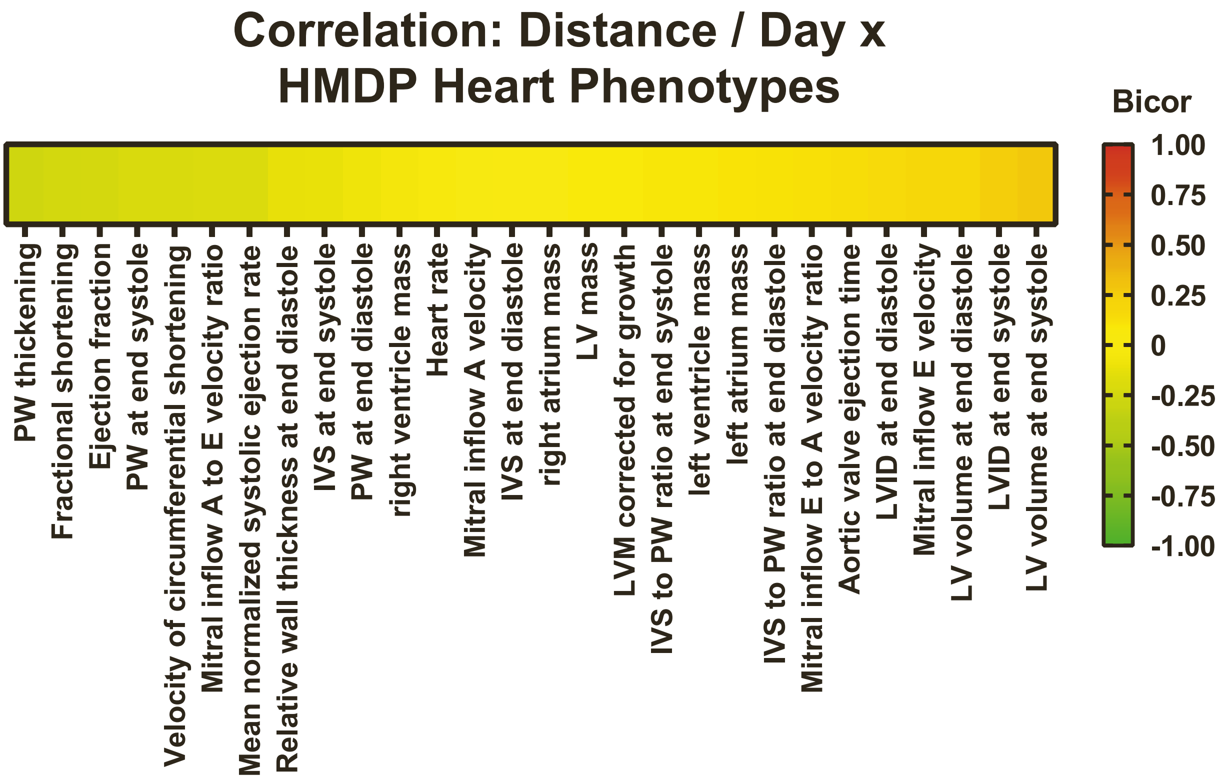

Moore et al, Supplemental Figure 3

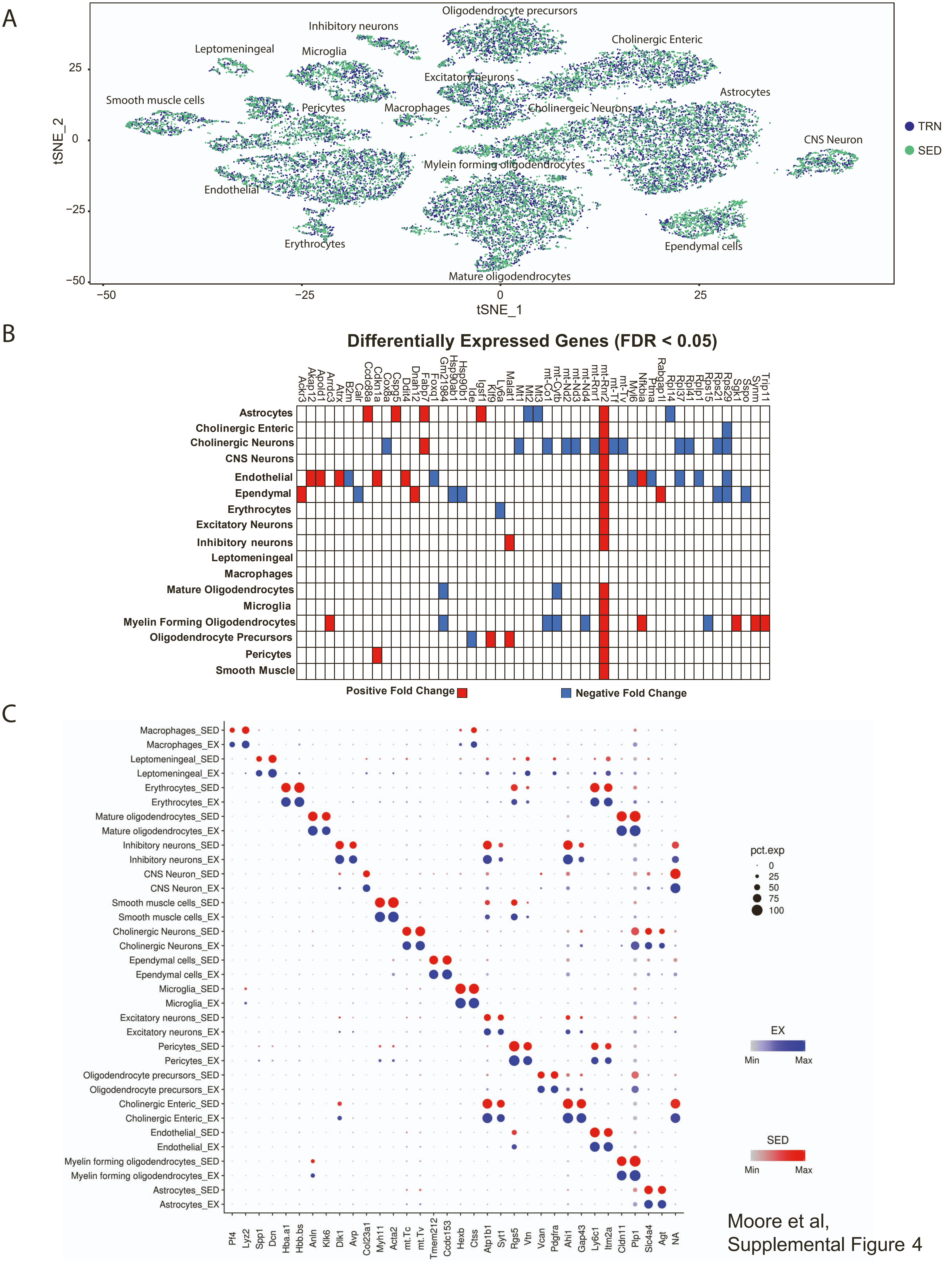

Moore et al,
Supplemental Figure 4

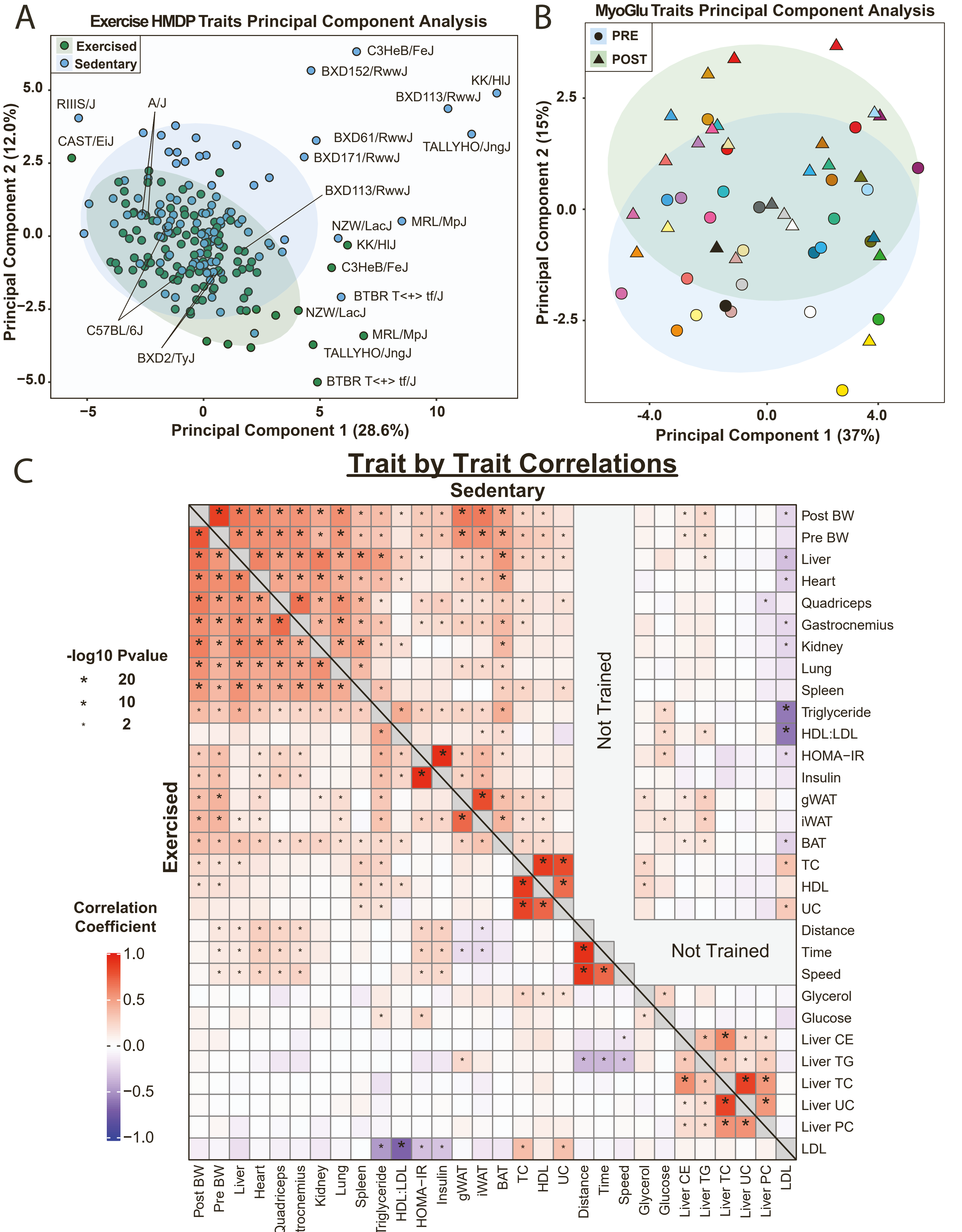

Moore et al, Supplemental Figure 5

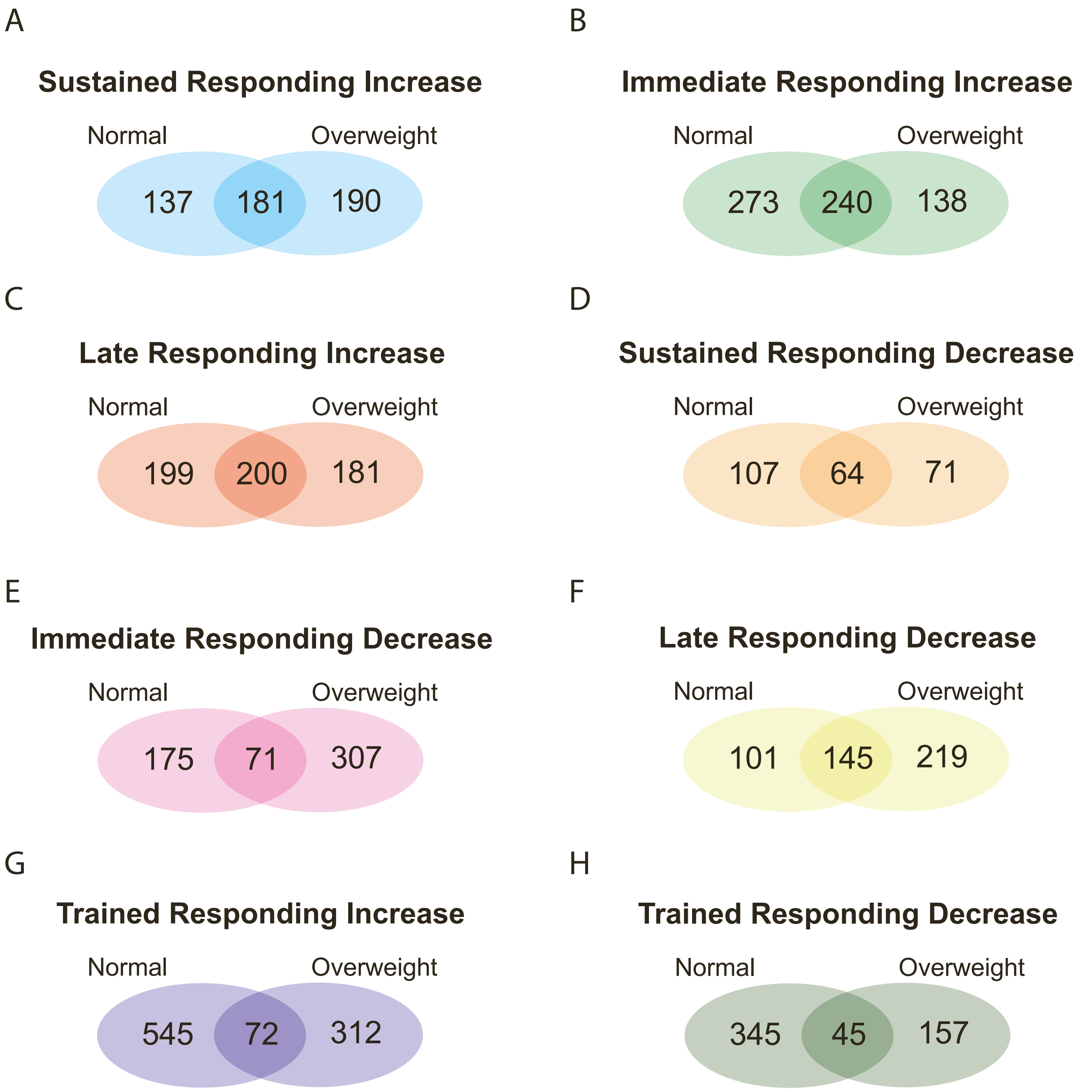

Moore et al, Supplemental Figure 6

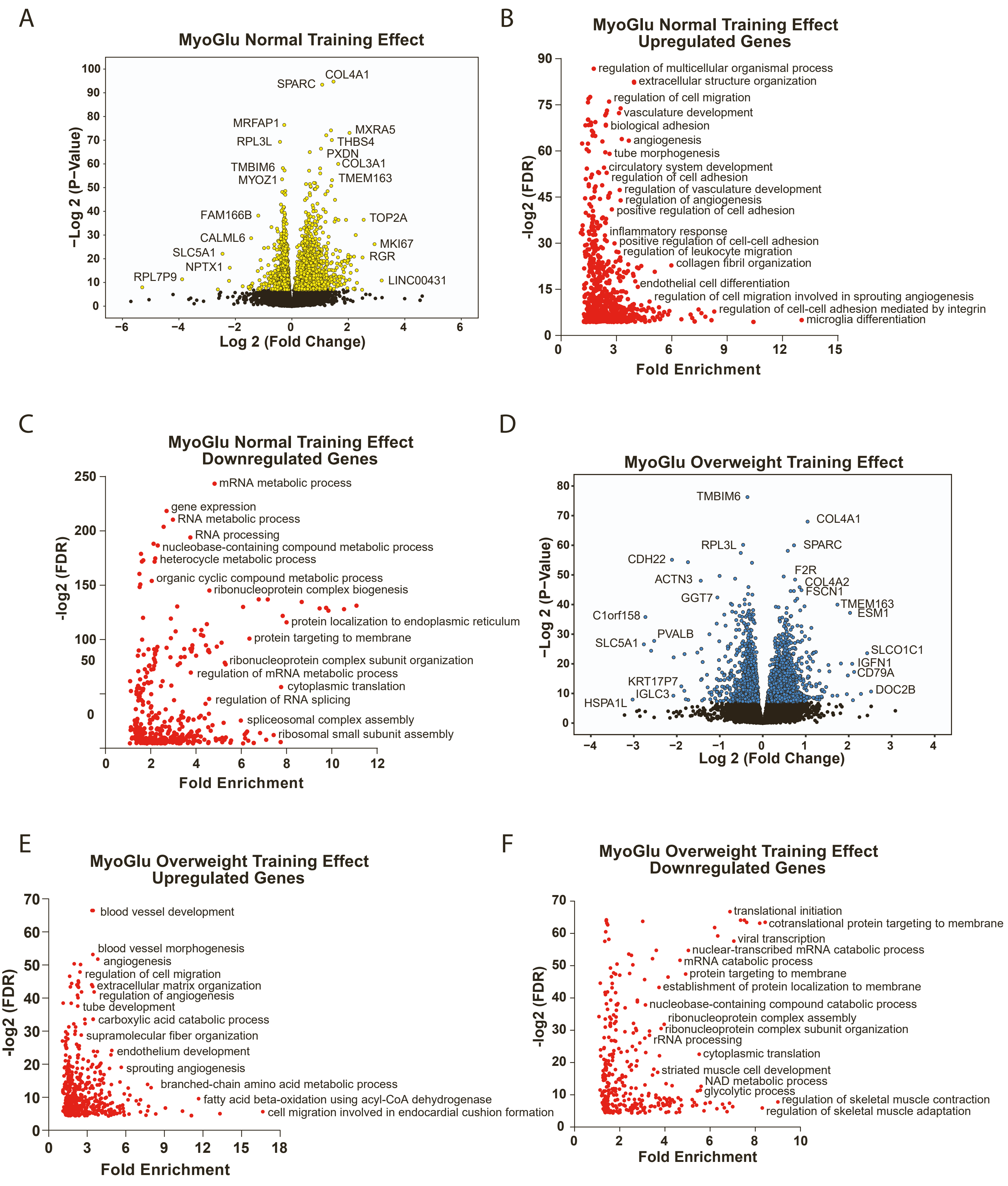

Moore et al, Supplemental Figure 7

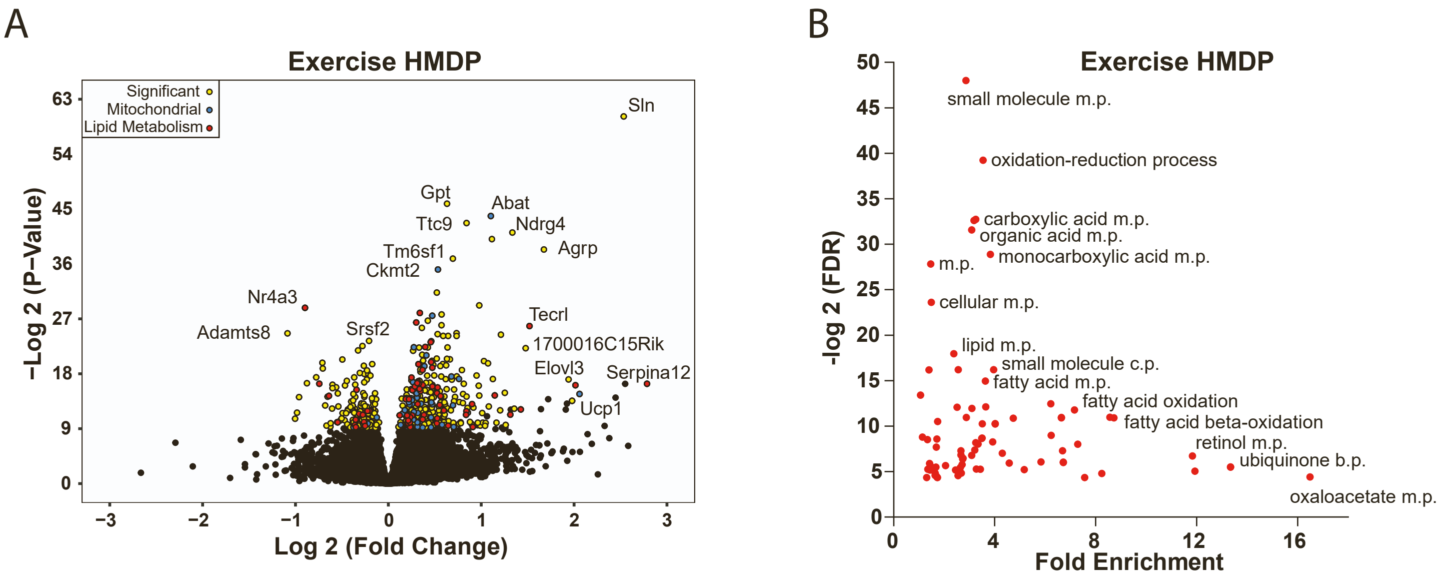

Moore et al, Supplemental Figure 8

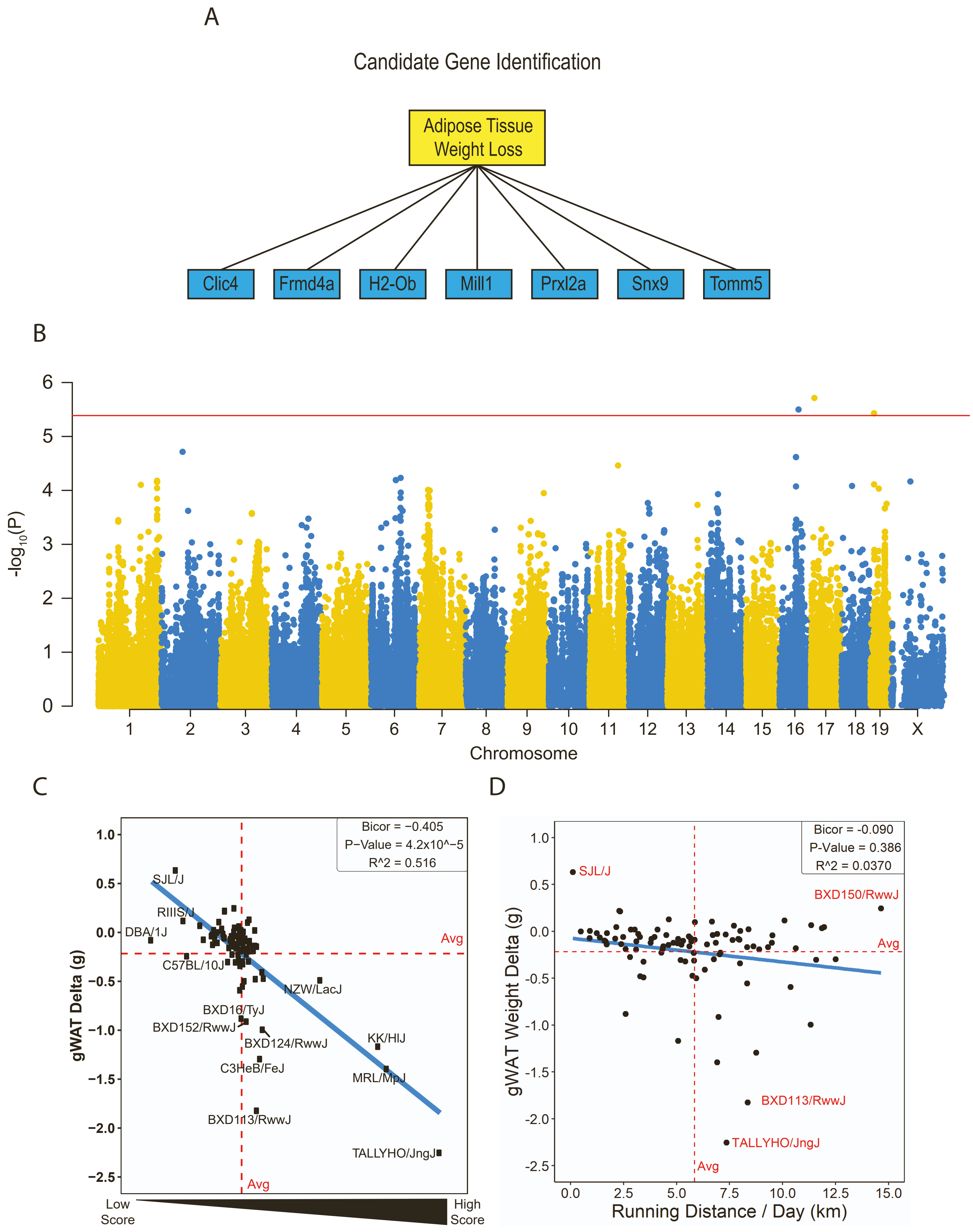

Moore et al, Supplemental Figure 9

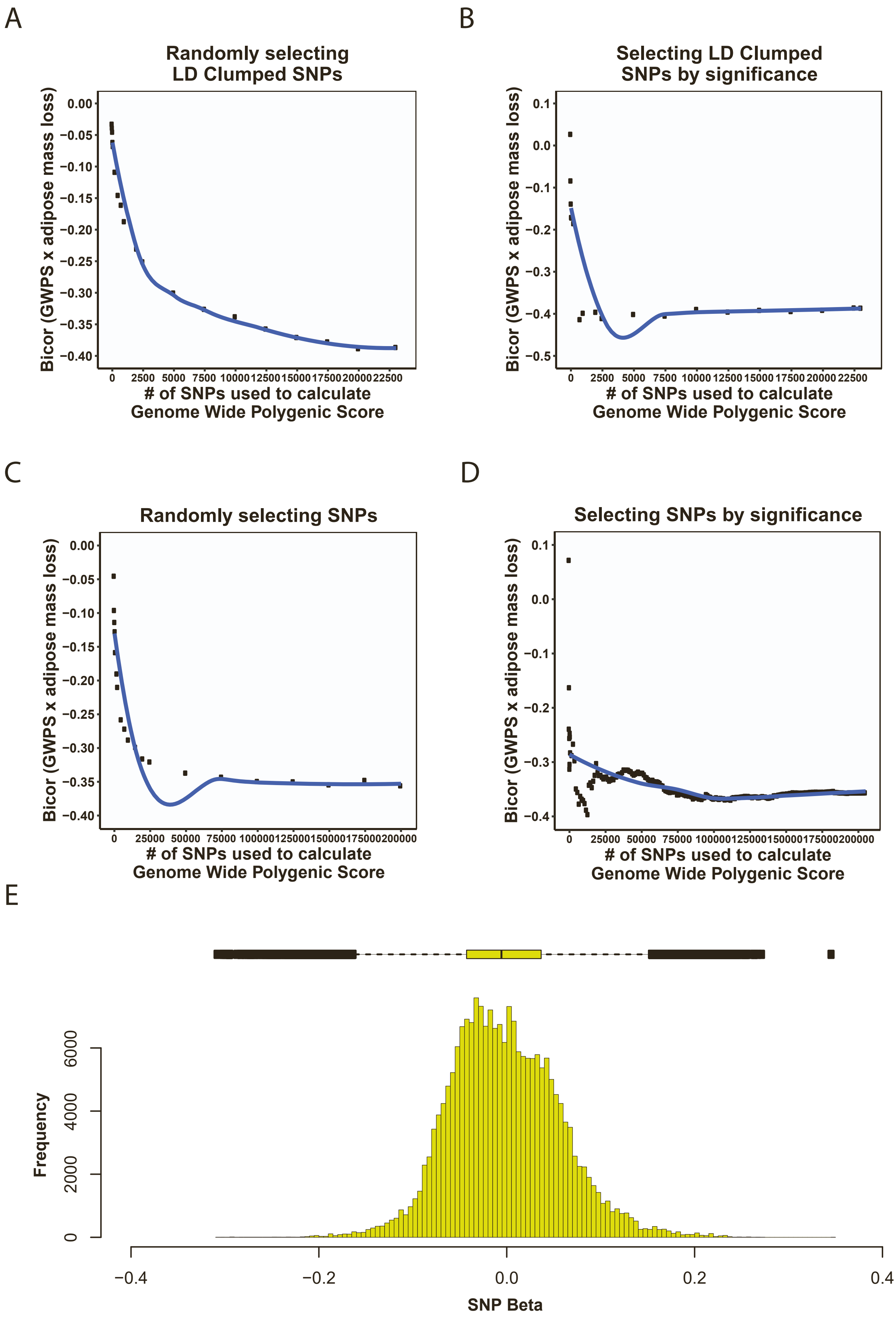

Moore et al, Supplemental Figure 10

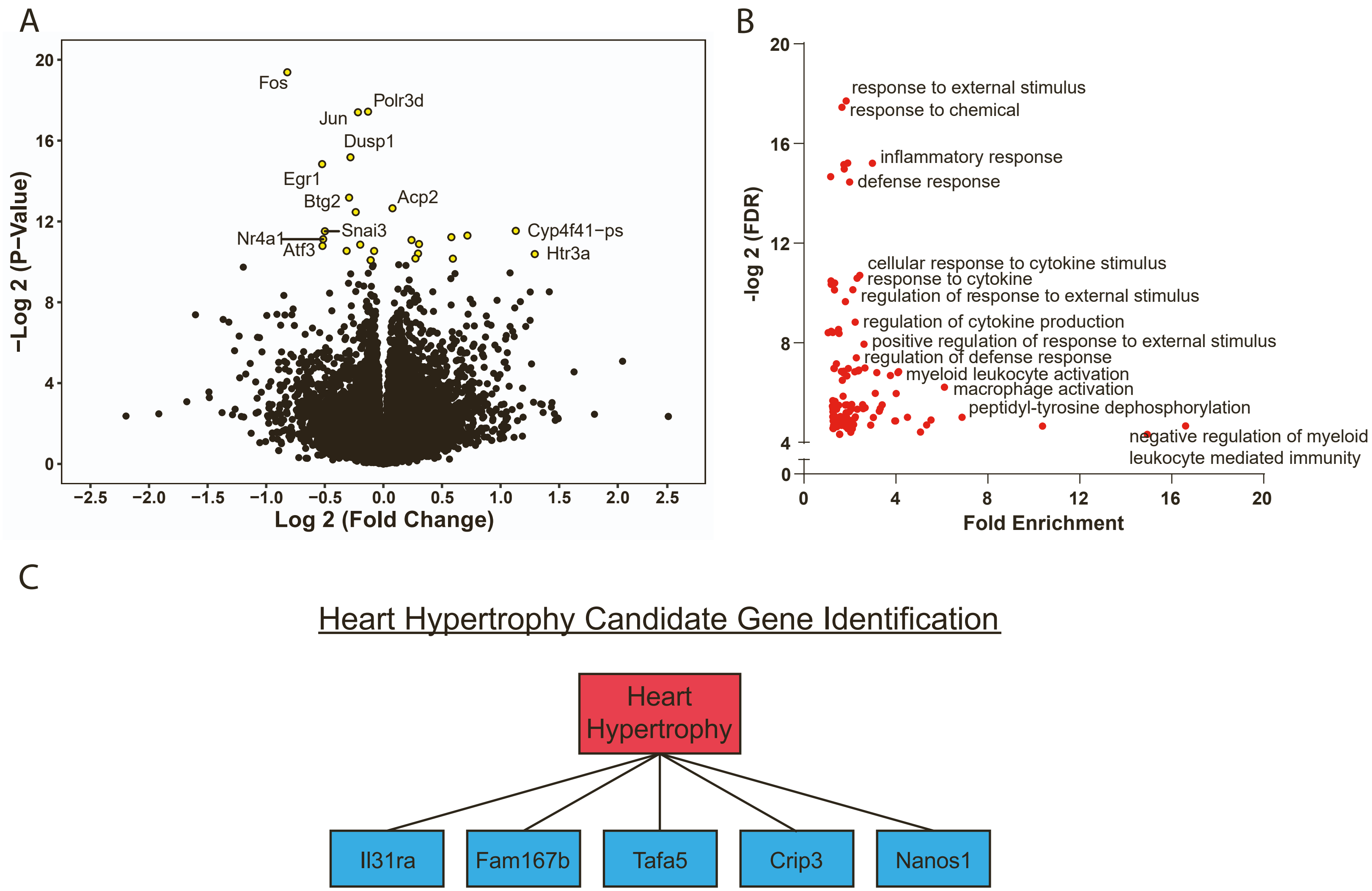

Moore et al, Supplemental Figure 11

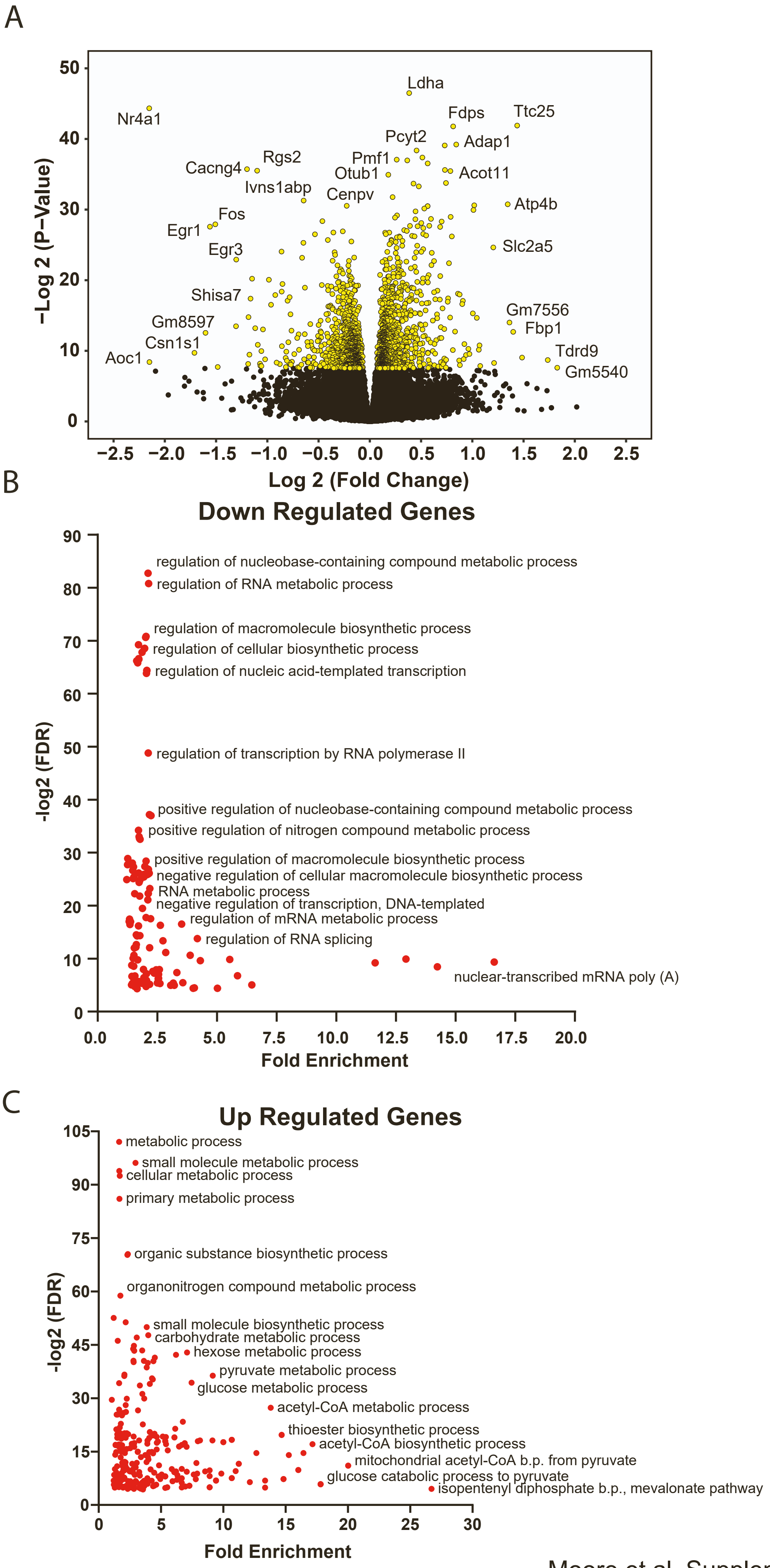

Moore et al, Supplemental Figure 12

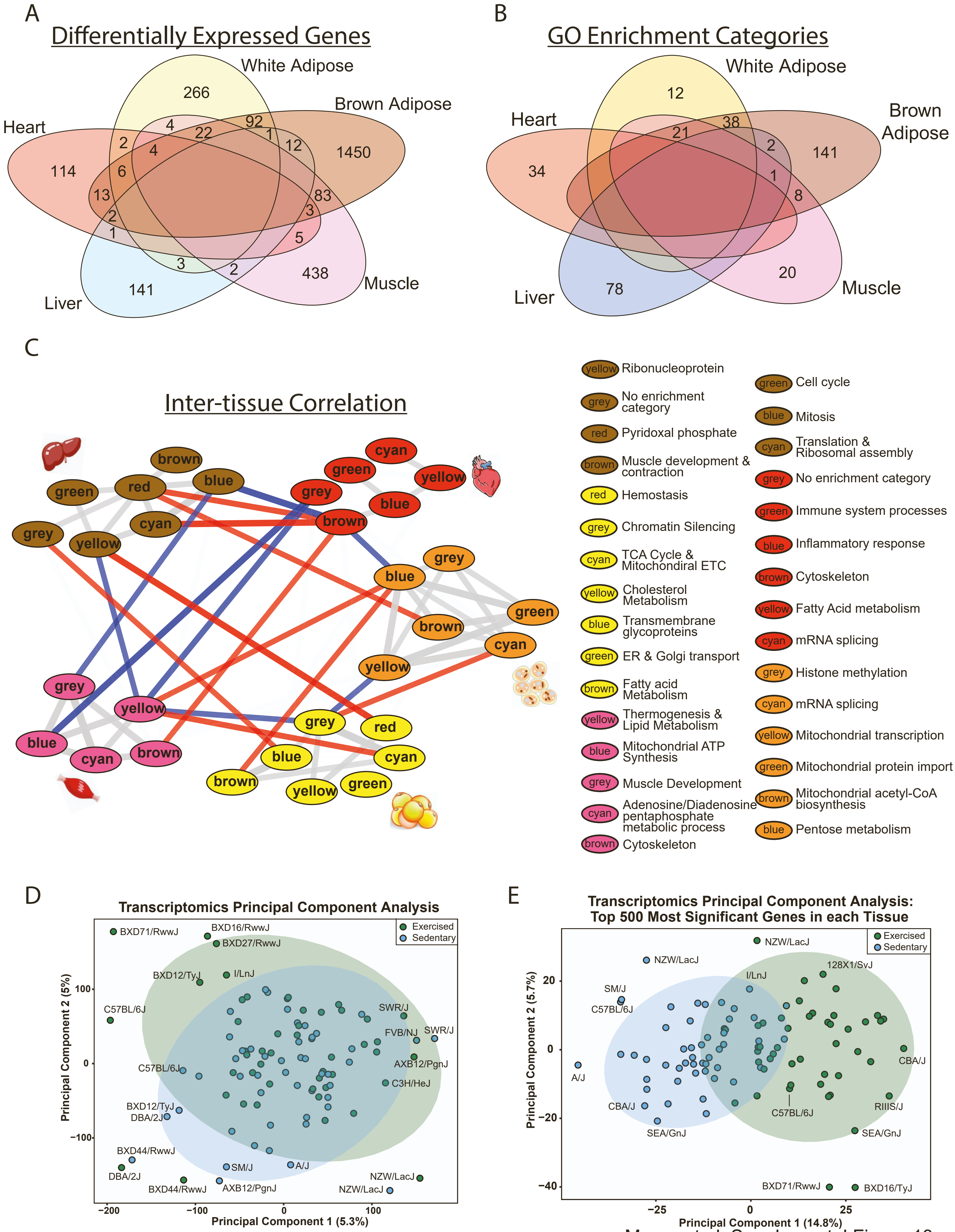

Moore et al, Supplemental Figure 13

Supplemental Figure Legends

Supplemental Figure 1. Overview of exercise intervention and tissue analysis in mice and humans (Associated with Figure 1)
(A) Female mice from the Hybrid Mouse Diversity Panel (HMDP) were exercise trained (TRN) on in-cage running wheels or remained sedentary (SED) for 30 days. Transcriptomics was performed on 9 tissues and correlated with clinical traits. (B) Transcriptomics was also performed on muscle and fat from normal weight and overweight individuals participating in the MyoGlu trial, where subjects performed both acute and long-term endurance exercise. (C) Data have been integrated and made publicly available for comparative analysis in a Shiny web application found at https://exchmdpmg.medsch.ucla.edu.

Supplemental Figure 2. Genome wide polygenic score analysis (Associated with Figure 1)
(A-E) Correlation where the x-axis represents the number of SNPs used to calculate the genome wide polygenic score (GWPS) for each strain and the y-axis represents the bicorrelation value between the strain average of the GWPS and the distance run per day either by selecting SNPs randomly or selecting SNPs based upon significance. Linkage disequilibrium (LD) clumped SNPs removed all SNPs with $r^2 > 0.6$. (E) Frequency distribution with box plot (above) indicating the distribution of values for Beta (effect size for each SNP).

Supplemental Figure 3. Comparative analyses of heart traits in two distinct HMDPs (Associated with Figure 1)
Correlation heatmap between average daily running distance (km) from the exercise HMDP and heart traits from the female heart failure HMDP.

Supplemental Figure 4. Hypothalamus single cell sequencing (Associated with Figure 1)
(A) tSNE plot showing cell by group isolated from the hypothalamus of exercised and sedentary C57BL/6J mice. (B) DEGs (exercise trained vs sedentary) with red and blue colored boxes indicate positive or negative fold change respectively. Colored boxes are significant at FDR < 0.05. White boxes are not significantly different. (C) Genes used to classify cells.

Supplemental Figure 5. Correlations and PCA (Associated with Figure 3)
(A) Principal component analysis of ExcHMDP using all measured traits. Strains colored by group with group confidence interval highlighted. (B) Principal component analysis of MyoGlu using traits from before (pre) or after (post) long-term exercise training. Points colored by individual with group confidence interval highlighted. (C) Correlation heatmap showing sedentary trait-by-trait correlations (above diagonal) and trait by trait correlations following exercise intervention (below diagonal). *, correlation significance ($P < 0.05$).

Supplemental Figure 6. MyoGlu skeletal muscle (Associated with Figure 4)
(A, D) Volcano plot of fold change and significance from MyoGlu skeletal muscle between pre and post exercise training in normal weight and overweight groups respectively.

Significant genes (FDR<0.05) are colored. (B, C, E, F). Gene enrichment analysis from panels A & D. Only significantly enriched groups are displayed (FDR<0.05).

Supplemental Figure 7. MyoGlu skeletal muscle (Associated with Figure 4)
(A-H) Overlaps of significant transcripts between normal weight and overweight skeletal muscle from MyoGlu. Colors match transcript patterns from Figure 4.

Supplemental Figure 8. ExcHMDP skeletal muscle (Associated with Figure 4)
(A) Volcano plot of fold change and significance from skeletal muscle of ExcHMDP. Significant transcripts (FDR<0.05) and gene ontology categories are colored. (B) Gene enrichment analysis from panel A. Only significantly enriched groups are displayed (FDR<0.05).

Supplemental Figure 9. ExcHMDP gWAT (Associated with Figure 5)
(A) Candidate gene (blue rectangles) identification analysis for adipose tissue weight loss (yellow rectangle). (B) GWAS for the difference in gWAT between SED and TRN from the ExcHMDP, solid line = significance threshold. (C) Strain averaged correlation of the change in gonadal white adipose tissue mass (gWAT Delta (g)) and genome wide polygenic score derived from same trait. Only using SNPs with between SNP $r^2 < 0.6$ and GWAS $P< 0.01$. Dashed lines = overall axis trait average. (D) Correlation between the change in gonadal white adipose tissue mass (gWAT Weight Delta (g)) and running distance per day (km) by strain. Average running distance and gWAT delta are indicated by dashed lines. Correlation statistics indicated in top left. Notable strains are indicated.

Supplemental Figure 10. ExcHMDP gWAT (Associated with Supplemental Figure 7)
(A-D) Correlation where the x-axis represents the number of SNPs used to calculate the genome wide polygenic score (GWPS) for each strain and the y-axis represents the bicorrelation value between the strain average of the GWPS and the gonadal white adipose tissue mass loss either by selecting SNPs randomly or selecting SNPs based upon significance. LD clumped SNPs removed all SNPs with $r^2 > 0.6$. (E) Frequency distribution with box plot above indicating the distribution of values for Beta (effect size for each SNP).

Supplemental Figure 11. ExcHMDP Heart (Associated with Figure 2)
(A) Volcano plot of gene expression in the heart across all strains relative to the sedentary group. Significant genes (FDR<0.05) are yellow. (B) Gene enrichment analysis of DEGs with particular groups labeled. Only significantly enriched groups are displayed (FDR<0.05). (C) Candidate gene (blue rectangles) identification analysis for heart hypertrophy (red rectangle).

Supplemental Figure 12. ExcHMDP BAT (Associated with Figure 2)
(A) Volcano plot of gene expression in brown adipose tissue across all strains relative to the sedentary group. Significant transcripts (FDR<0.05) are yellow. (B-C) Gene enrichment analysis of DEGs for downregulated vs upregulated transcripts for trained vs. sedentary. Only significantly enriched groups are displayed (FDR<0.05).

Supplemental Figure 13. Integrated tissue analysis

(A) Venn diagram showing the overlap of DEG in muscle, heart, adipose, and liver. All gene significant at FDR<0.05 for skeletal muscle and brown adipose tissue or P < 0.001 for heart, liver, and gonadal white adipose tissue. Select genes annotated with text. (B) Venn diagram showing the overlap of Gene Ontology enrichment analysis in skeletal muscle, heart, gonadal white adipose tissue, brown adipose tissue, and liver. All GO Terms significant at FDR<0.05. (C) Correlation of modules derived from DEG within each tissue. Edge thickness indicates strength of correlation. Non-significant edges are not shown. Only significant inter-tissue module correlations are colored. Red edge = Positive correlation. Blue edge = negative correlation. Grey edges are significant within a tissue. Node label indicates tissue specific module name. Node color indicates tissue. Red = heart. Yellow = gonadal white adipose tissue. Pink = muscle. Brown = liver. Orange = brown adipose tissue. Outside node text indicates top gene enrichment category for module. (D-E) Principal component analysis using all transcriptomics data or top 500 most significant transcripts from all tissues (Liver, gWAT, Heart, BAT, QUAD). Strains colored by group.
